# p120 Catenin-Mediated Stabilization of E-Cadherin Is Essential for Primitive Endoderm Specification

**DOI:** 10.1371/journal.pgen.1006243

**Published:** 2016-08-24

**Authors:** Tim Pieters, Steven Goossens, Lieven Haenebalcke, Vanessa Andries, Agata Stryjewska, Riet De Rycke, Kelly Lemeire, Tino Hochepied, Danny Huylebroeck, Geert Berx, Marc P. Stemmler, Dagmar Wirth, Jody J. Haigh, Jolanda van Hengel, Frans van Roy

**Affiliations:** 1 Department of Biomedical Molecular Biology, Ghent University, Ghent, Belgium; 2 Inflammation Research Center, VIB, Ghent, Belgium; 3 Center for Medical Genetics, Ghent University Hospital, Ghent, Belgium; 4 Cancer Research Institute Ghent (CRIG), Ghent, Belgium; 5 Department of Development and Regeneration, Laboratory of Molecular Biology (Celgen), University of Leuven, Leuven, Belgium; 6 Department of Cell Biology, Erasmus University Medical Center, Rotterdam, The Netherlands; 7 Department of Molecular Embryology, Max-Planck Institute of Immunobiology, Freiburg, Germany; 8 Department of Experimental Medicine I, Nikolaus-Fiebiger Center for Molecular Medicine, University of Erlangen-Nürnberg, Erlangen, Germany; 9 Helmholtz Center for Infection Research, Braunschweig, Germany; 10 Mammalian Functional Genetics Laboratory, Division of Blood Cancers, Australian Centre for Blood Diseases, Department of Clinical Haematology, Monash University and Alfred Health Alfred Centre, Melbourne, Victoria, Australia; 11 Department of Basic Medical Sciences, Faculty of Medicine and Health Sciences, Ghent University, Ghent, Belgium; Institut Curie, FRANCE

## Abstract

E-cadherin-mediated cell-cell adhesion is critical for naive pluripotency of cultured mouse embryonic stem cells (mESCs). E-cadherin-depleted mESC fail to downregulate their pluripotency program and are unable to initiate lineage commitment. To further explore the roles of cell adhesion molecules during mESC differentiation, we focused on p120 catenin (p120ctn). Although one key function of p120ctn is to stabilize and regulate cadherin-mediated cell-cell adhesion, it has many additional functions, including regulation of transcription and Rho GTPase activity. Here, we investigated the role of mouse p120ctn in early embryogenesis, mESC pluripotency and early fate determination. In contrast to the E-cadherin-null phenotype, p120ctn-null mESCs remained pluripotent, but their *in vitro* differentiation was incomplete. In particular, they failed to form cystic embryoid bodies and showed defects in primitive endoderm formation. To pinpoint the underlying mechanism, we undertook a structure-function approach. Rescue of p120ctn-null mESCs with different p120ctn wild-type and mutant expression constructs revealed that the long N-terminal domain of p120ctn and its regulatory domain for RhoA were dispensable, whereas its armadillo domain and interaction with E-cadherin were crucial for primitive endoderm formation. We conclude that p120ctn is not only an adaptor and regulator of E-cadherin, but is also indispensable for proper lineage commitment.

## Author Summary

Disease may be due to either excess of undesirable cells, like in cancer or autoimmune disease, or by progressive loss of vital cells. The latter, for instance, causes neurodegenerative diseases such as Alzheimer’s disease. Stem-cell based therapy holds great potential to cure devastating diseases with cell loss or dysfunctionality, because stem cells have the capacity to form any given cell type of the body. Recent advances in the field allow to obtain stem cells from virtually every patient. These stem cells could then be instructed to form the desired cells that can be reintroduced to cure the patient. Before such therapies are suited for the clinic, we first need comprehensive knowledge of the molecular mechanisms that underlie cell fate decisions. Here, we scrutinize the role of a junctional protein, called p120ctn, in both stem cells and lineage-committed cells. Importantly, this key protein has a modular structure, and each of its segments has different interaction partners and biological functions. We deleted p120ctn specifically in stem cells, and reintroduced several p120ctn mutants that lack specific protein segments. As such, we could unravel the exact molecular interaction that is required for p120ctn to drive the differentiation of stem cells towards primitive endoderm.

## Introduction

Pluripotent mouse embryonic stem cells (mESCs) can self-renew and differentiate into any given cell type within an organism. They are isolated from the inner cell mass (ICM) of preimplantation blastocyst stage embryos and considered ‘naive’, with regard to their pluripotency status, whereas stem cells derived from the epiblast of the post-implantation embryo are considered ‘primed’ [1]. Naive mESCs can be maintained in a ‘ground’ state when they are cultured in LIF-containing medium containing two small-molecule inhibitors (2i) directed against, respectively, Erk and Gsk3 [2]. E-cadherin is a critical regulator of naive pluripotency [3], as its genetic inactivation in mESCs facilitates them to convert from a naive to a primed pluripotency state [4]. In addition, E-cadherin is required for proper compaction between the blastomeres of the morula stage embryo and for subsequent trophectoderm formation during the first cell fate segregation decision in embryos [5, 6]. As E-cadherin-null embryos do not form proper blastocysts, the role of E-cadherin during mouse embryogenesis beyond this stage remains elusive.

Early stages of mouse embryogenesis can be recapitulated *in vitro* by allowing pluripotent mESCs to differentiate into three-dimensional cell aggregates grown in suspension, known as embryoid bodies (EBs) [7]. Key signals such as BMPs, Wnt and Activin/Nodal can instruct EBs to produce specific descendants from all three germ layers [8]. EB formation mimics embryonic development because mESC aggregates resemble morula-like structures, whereas cystic EBs resemble the blastocyst or egg-cylinder stage. Indeed, the blastocyst consists of the inner ectodermal layer, which will form the epiblast, and a surrounding layer of visceral endoderm (VE), which is derived from the ICM-derived primitive endoderm or hypoblast of the blastocyst [9]. The VE is marked by α-fetoprotein (AFP) and E-cadherin [10, 11].

E-cadherin, encoded by *Cdh1*, is a transmembrane protein that mediates homophilic calcium-dependent cell–cell adhesion via its extracellular domains. Its cytoplasmic tail contains two highly conserved domains to which the armadillo proteins β-catenin and p120ctn respectively bind [12]. β-catenin is a bifunctional molecule: it is part of the cadherin/catenin cell adhesion complex and it has a key transactivating role in the Wnt signaling pathway. Both roles seem to be important for naive pluripotency [13–15].

p120ctn is also a multi-functional protein [16]. It binds to the juxtamembrane domain of classical cadherins, including E-cadherin, and thereby stabilizes them by preventing their endocytosis [17]. In addition, p120ctn can modulate the activities of Rho GTPase family members [18]. p120ctn contains two RhoA binding domains (RBD1 and RBD2), which are important for modulating RhoA activity. Deleting RBD2 (amino acids (AA) 622–628 in mouse p120ctn) abolishes the p120ctn-mediated inhibition of RhoA activity [19] and deleting both RBDs completely disrupts the interaction of p120ctn with RhoA [20]. The RBD2 sequence has also been identified as a nuclear localization sequence (NLS) [21]. Finally, p120ctn participates in gene transcription by alleviating Kaiso- and RESR/coREST-mediated repression on their respective target genes [22, 23].

As a result of alternative splicing, p120ctn isoforms can be generated from four alternative start codons [24]. p120ctn isoform 1A is the longest as it is translated from the most 5’ start codon and is composed of a centrally located armadillo domain flanked by an N- and a C-terminal domain. The extended N-terminal segment comprises several functional domains and confirmed phosphorylation sites [16, 25]. In p120ctn isoforms 3 and 4, this N-terminal segment is deleted partially and completely, respectively. The long and short p120ctn isoforms (1A and 3A, respectively) display specific expression depending on the cell type and have opposing functions in particular human cancers [26, 27]. The functions of p120ctn in mESC biology and the contribution of its many isoforms and interaction domains remain unknown.

Our aim was to dissect at the molecular level, the precise role of p120ctn in naive pluripotency and lineage commitment of mESCs. We show that p120ctn-null EBs cannot form primitive endoderm and identify the crucial mediator of this process using a structure-function approach. Key to our approach was the introduction of a large panel of natural p120ctn variants and mutants, one by one, into p120ctn-deficient mESCs via recombinase-mediated cassette exchange (RMCE). We demonstrate that p120ctn-mediated stabilization of E-cadherin is required for differentiation of mESCs towards well-polarized primitive endoderm.

## Results

### E-cadherin is required for mESC lineage commitment

**Fig 1:**
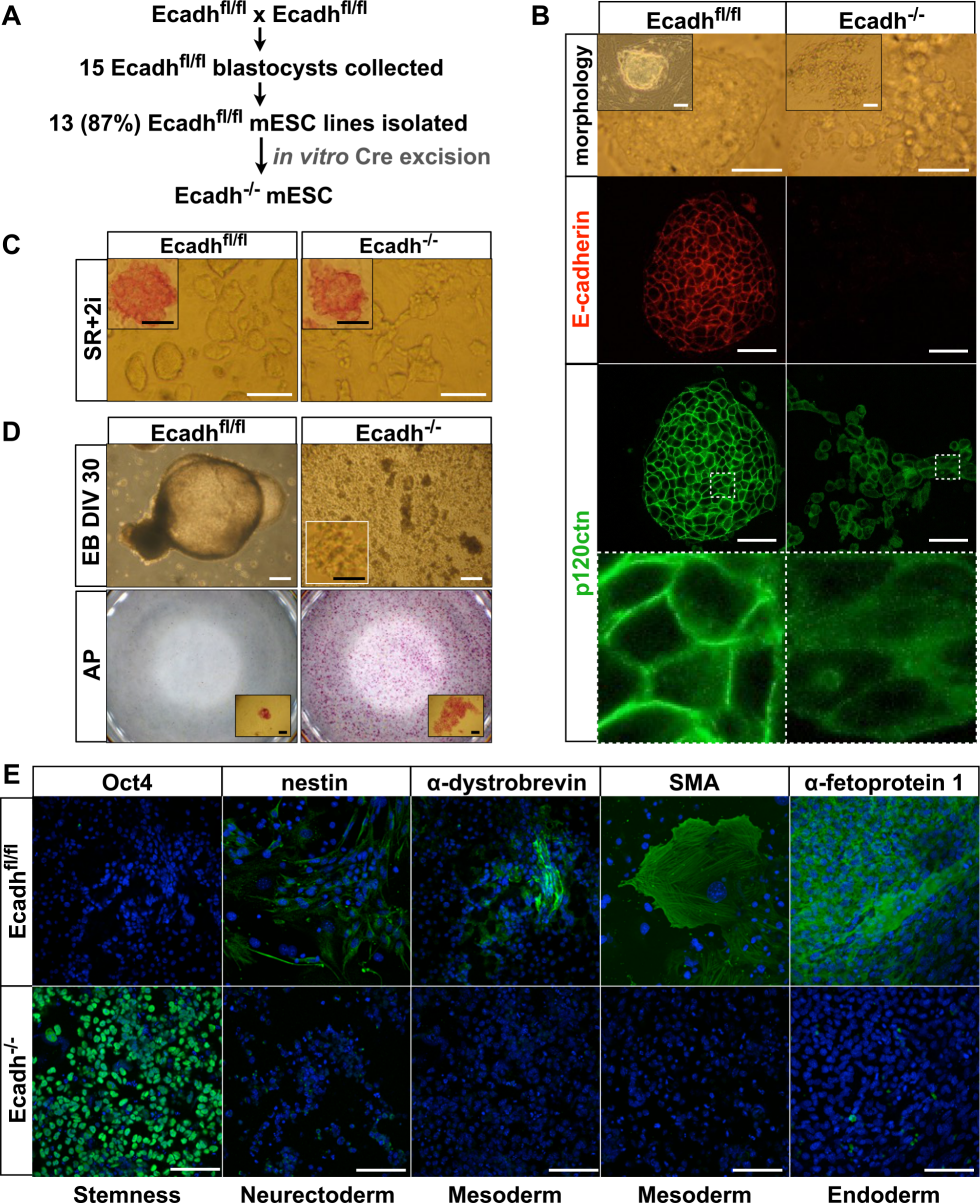
E-cadherin-null mESCs fail to maintain cellular aggregation and show defects in differentiation. **(A)** Scheme of the isolation and Cre-mediated recombination of Ecadh^fl/fl^ mESCs leading to E-cadherin-null mESCs. **(B)** Morphology and immunostaining for E-cadherin and p120ctn of E-cadherin-floxed (Ecadh^fl/fl^, control) and E-cadherin-null (Ecadh^-/-^) mESCs that were cultured on mitomycin-C-treated mouse embryonic fibroblasts (MEFs) and in serum-containing medium. E-cadherin loss resulted in defective mESC aggregation and in cytoplasmic p120ctn levels. An eight-fold magnification of the boxed area is shown in the bottom panels. Scale bars: 50 μm. **(C)** Morphology and alkaline phosphatase (AP) staining of control and E-cadherin-null mESCs that were cultured in SR-mESC medium supplemented with 2i. White scale bars: 100 μm. Black scale bars: 25 μm. **(D)** Embryoid bodies (EBs) from control and E-cadherin-null mESCs were cultured for 30 days *in vitro* (DIV30, top panel). Thereafter, EBs were trypsinized, and an equal number of dissociated cells was cultured for 4 days in SR-mESC medium and then stained for AP (bottom panel). Inset: morphology of dissociated EB cells after replating and AP staining. E-cadherin-null mESCs failed to form EBs and retained AP activity. White scale bars: 200 μm. Black scale bars: 100 μm. **(E)** Fluorescent images showing control and E-cadherin-null EB-derived cells that were plated for 10 days and then stained for Oct4 (stem cell marker), nestin (neurectoderm), skeletal muscle marker α-dystrobrevin (mesoderm), smooth muscle actin (SMA, mesoderm) and α-fetoprotein 1 (endoderm). Cultures derived from E-cadherin-null mESCs failed to exit their self-renewal and did not commit towards differentiation. Scale bars: 100 μm.

Using a pluripotin-based protocol [28], we isolated mESCs with floxed *Cdh1* alleles (Fig 1A), referred hereafter as control mESCs. These cells were subsequently subjected to Cre-mediated recombination *in vitro* to obtain E-cadherin-null mESC lines. As previously reported, these mESCs have cell-cell adhesion defects, making classic isolation and maintenance of these cells difficult (Fig 1B) [4, 5]. However, we achieved to serially passage them with the use of 2i-supplemented serum replacement (SR)-mESC medium (Fig 1C). While control mESCs formed cystic EBs, E-cadherin-null mESCs formed only loose cell aggregates, but no cystic EBs (Fig 1D, top panel). Even after a culture period of 30 days under differentiation conditions, E-cadherin-null cells retained full ability to form alkaline phosphatase (AP)-positive colonies when shifted back to naive mESC culture conditions (Fig 1D, bottom). In addition, all the E-cadherin-deficient cells analyzed remained positive for the stem cell marker Oct4 and negative for specific markers of each of the three germ layers (Fig 1E), as demonstrated by immunostaining. This is in agreement with previous findings [29, 30]. Thus, E-cadherin is essential for lineage commitment of mESCs.

### Loss of p120ctn does not impede early embryogenesis

Upon p120ctn loss, cell surface E-cadherin is internalized and degraded [17, 31, 32]. To scrutinize the roles of E-cadherin in later stages of mESC differentiation, we studied its interrelation with the armadillo protein p120ctn in the context of mESCs and early mouse embryogenesis.

**Fig 2:**
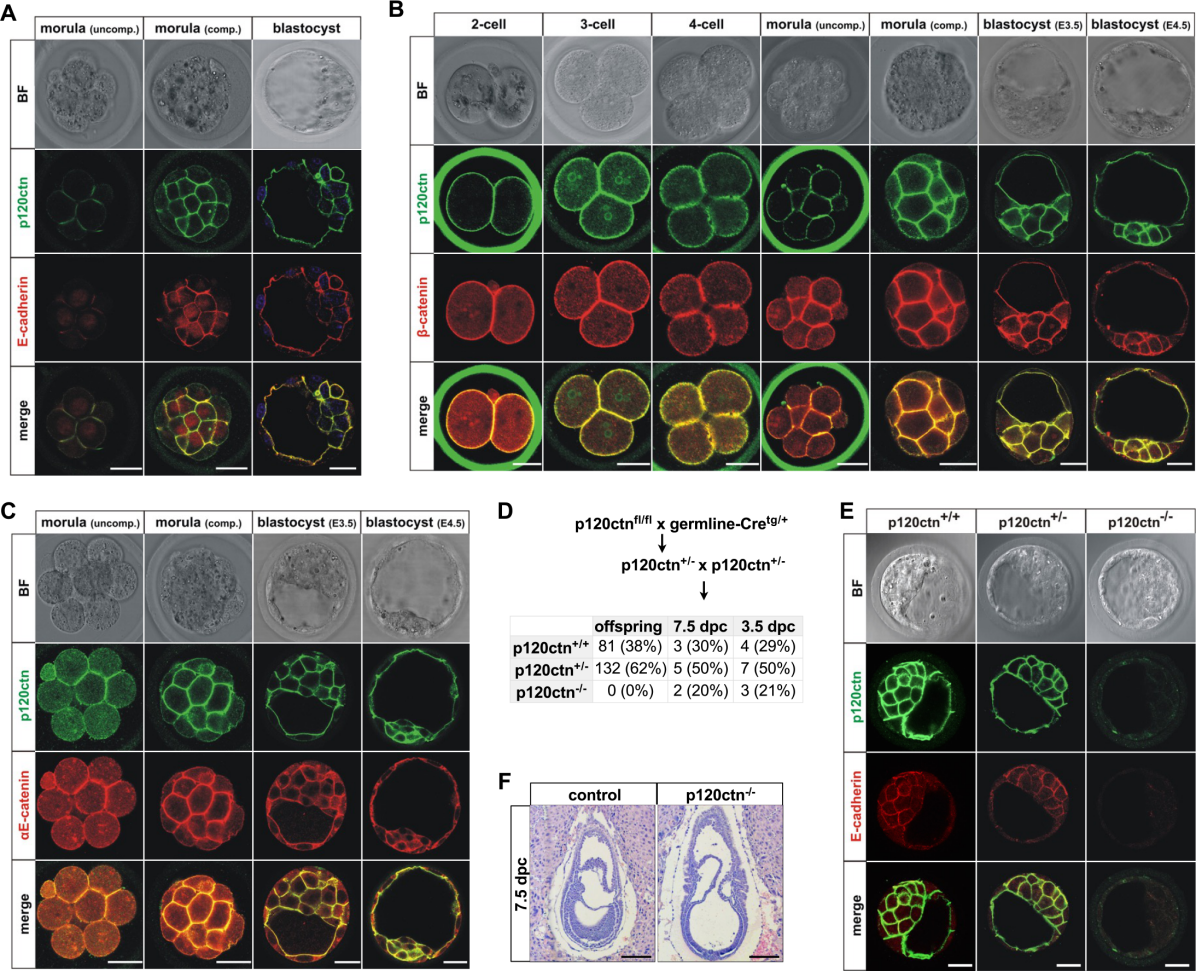
Early embryogenesis occurs normally in p120ctn-depleted embryos. **(A-C)** p120ctn co-localizes with cadherin-based junctional complexes in wild-type preimplantation mouse embryos. Bright field (BF) transmitted light micrographs and immunofluorescence of wild-type embryos from the two-cell stage to blastocysts, including uncompacted (uncomp.) and compacted (comp.) morulas. Double immunofluorescence was done for p120ctn (green signal) and for E-cadherin **(A)**, αE-catenin **(C)** or β-catenin **(B)**. Scale bars: 25 μm. **(D)** Breeding scheme to obtain p120ctn^-/-^ embryos in timed matings. The table depicts numbers and percentages of p120ctn^+/+^, p120ctn^+/-^ or p120ctn^-/-^ embryos that were recovered at the developmental stages indicated (dpc, days post coitum). **(E)** BF micrographs and immunostainings of 3.5-dpc blastocysts of wild-type (p120ctn^+/+^) mice, heterozygous (p120ctn^+/-^) and homozygous (p120ctn^-/-^) p120ctn knock-out mice. Double immunofluorescence was performed for p120ctn and E-cadherin. Scale bars: 25 μm. **(F)** Hematoxylin and eosin (H&E)-stained paraffin sections of control and p120ctn^-/-^ gastrula-stage embryo. Scale bars: 200 μm.

First, we analyzed the expression of p120ctn during early mouse development. We found that p120ctn was co-expressed with E-cadherin at the basolateral membranes of mouse blastocysts (Fig 2A, S1A Fig). Throughout the preimplantation phases, p120ctn co-localized with the junctional proteins E-cadherin, α-catenin and β-catenin, and is enriched at the surface of morula stage cells from the start of compaction onwards (Fig 2A–2C). Next, we investigated the role of p120ctn during embryonic development by genetic inactivation of p120ctn. We used mice, in which exons 3 to 8 of the p120ctn gene (*Ctnnd1)* were floxed (hereafter referred to as control mice) [33]. Germline Cre-mediated recombination resulted in the generation of heterozygous p120ctn knockout mice. As previously published, no viable homozygous p120ctn-null mice were recovered (Fig 2D) [33, 34]. Remarkably, unlike E-cadherin loss, genetic inactivation of p120ctn did not affect blastocyst formation, although E-cadherin levels were drastically reduced in such p120ctn-null blastocysts (Fig 2E). Only a small amount of membrane-localized E-cadherin is required for morula compaction *in vivo*, as maternal E-cadherin allows compaction in E-cadherin-deficient embryos [5]. We detected a small amount of p120ctn staining above the background level, indicative of maternal p120ctn protein (S1B Fig). This maternal p120ctn probably allows sufficient membrane expression of E-cadherin in p120ctn^-/-^ embryos (S1B Fig, arrowheads) to mediate morula compaction. This implies that p120ctn expression is the rate limiting factor for E-cadherin expression in early embryos. p120ctn-null embryos could still be recovered in the expected Mendelian ratio at E7.5 (Fig 2D and 2F). So, in contrast to E-cadherin-null embryos, loss of p120ctn does not abrogate early embryonic development.

### Loss of p120ctn induces a mild differentiation defect in cultured mESC

**Fig 3:**
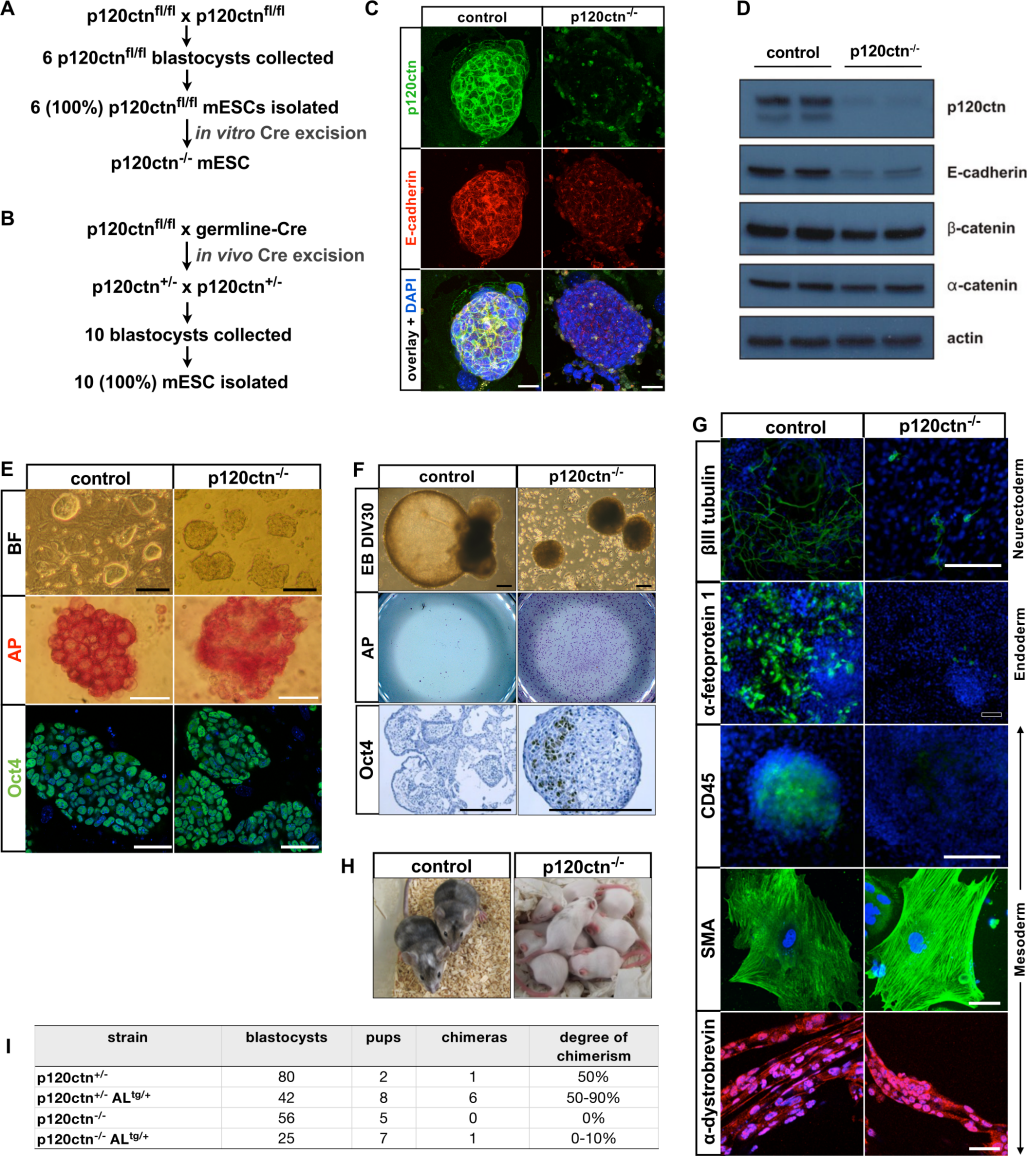
p120ctn loss affects junctional stability of mESCs, has no effect on self-renewal but partly inhibits differentiation of mESCs. **(A)** Scheme of isolation of p120ctn^fl/fl^ mESCs (referred to as control) followed by *in vitro* Cre-mediated recombination. **(B)** Scheme of mouse breedings that allow germline Cre-mediated recombination of p120ctn^fl/fl^ mice *in vivo*, followed by the isolation of p120ctn^+/+^ and p120ctn^+/-^ mESCs (both referred to as control), and p120ctn^-/-^ mESCs. **(C)** Confocal fluorescent images of control and p120ctn-null mESCs stained for p120ctn and E-cadherin. Scale bars: 25 μm. **(D)** Western blot analysis of two control (p120ctn^fl/fl^) and two p120ctn-null mESC lines. Antibodies used were specific for junctional components as indicated. Actin was used as loading control. **(E)** Morphology, alkaline phosphatase (AP) activity, and Oct4 immunostaining of control and p120ctn-null mESCs. Black scale bars: 100 μm, white scale bars: 50 μm. **(F)** Control and p120ctn-null EBs were cultured for 30 days *in vitro* (DIV30, top panels). Thereafter, they were trypsinized, cultured for 4 days in SR-mESC medium, and stained for AP (middle panels). Oct4 immunohistochemistry was performed on paraformaldehyde-fixed paraffin sections of control and p120ctn-null EBs (DIV30, bottom panels). The experiment was performed with two independent control and p120ctn-null mESC lines and was reproduced twice. Scale bars: 200 μm. **(G)** Fluorescent images showing control and p120ctn-null EB-derived cells, which were plated for 10 days and then stained for a neurectodermal marker (βIII-tubulin), an endodermal marker (α-fetoprotein 1), and three mesodermal markers (CD45, smooth muscle actin/SMA, α-dystrobrevin). This differentiation experiment was performed twice. White scale bars: 200 μm; black scale bar: 200 μm. Pictures **(H)** and overview table **(I)** of offspring mice, obtained by diploid embryo aggregation assays with control and p120ctn-null mESCs.

To evaluate the functional importance of p120ctn and E-cadherin co-presence at the cell surface of mESCs (Fig 1B), we derived several control and p120ctn-null mESCs, either by *in vitro* Cre recombination of isolated p120ctn floxed mESCs (Fig 3A), or by *in vivo* Cre recombination followed by mESC derivation (Fig 3B). All p120ctn-null lines had normal karyotypes (S2A and S2B Fig) and had reduced E-cadherin levels (Fig 3C and 3D). Due to the loss of E-cadherin at the cell surface, the membrane-associated docking sites for catenins were lost as well. Only a minor effect on the expression and localization of α- and β-catenin was observed upon p120ctn loss (Fig 3D and S2C Fig). This indicates that in these cells the integrity of the entire cadherin–catenin complex is affected. Strikingly, even with partly dismantled cadherin-based junctions, p120ctn-null mESCs still formed compact colonies (Fig 3E).

We then analyzed the expression of stem cell marker genes in both control and p120ctn-depleted mESCs, but no differences were seen (Fig 3E, S2D Fig). To conclude, although the loss of p120ctn led to a reduction of E-cadherin levels, it did affect neither the stemness nor the typical dome-shaped morphology of mESC colonies.

To further test the effect of p120ctn loss on the differentiation capacity of mESCs, we subjected the cells to an EB differentiation protocol. Initially (3 to 5 days of *in vitro* culture), both control and p120ctn-null mESC lines generated EBs with a basic morphology consisting of a core of ectodermal and/or mesodermal cells covered with an outer layer of endoderm-like cells. This indicates that loss of p120ctn did not impair cellular aggregation during initial EB formation. From 7 days *in vitro* (DIV7), the control EBs started to develop as cystic EBs. However, p120ctn-depleted mESCs failed to form EBs with a cystic appearance, not even after 30 days (Fig 3F). Remarkably, such DIV30 cultured p120ctn-depleted EBs maintained their ability to form undifferentiated AP-positive mESC colonies after replating, whereas control cells did not (Fig 3F). Oct4-positive cells were detected in p120ctn-deficient EBs, but not in control cystic EBs, which were already terminally differentiated and therefore lost Oct4 expression (Fig 3F, S2E Fig). In line with these results, p120ctn-deficient EBs (DIV30) also failed to downregulate Sox2 and Eras (S2E Fig).

To document the differentiation phenotype of p120ctn-null mESCs, we carried out marker analysis on EBs and we also generated teratomas as well as chimeric mice from control and p120ctn-null mESCs. First, we performed EB differentiation experiments *in vitro*. Based on immunostainings of EBs, we concluded that control mESC produced cell types descending from each germ layer, including large fields of βIII-tubulin-positive neurons (neurectoderm), AFP-positive cells (endodermal like cells), CD45-positive hematopoietic cells, and α-dystrobrevin- and SMA-positive muscle cells (mesoderm) (Fig 3G). In contrast, only few p120ctn-null cells differentiated into these various cell types, although some markers, such as SMA and α-dystrobrevin, stained positive in p120ctn-null cells (Fig 3G). Second, upon injection of p120ctn-null mESCs into immunocompromised mice, we observed the formation of well-differentiated teratomas (S3 Fig). However, endoderm-related cells/structures were observed less frequently in the p120ctn-null teratomas.

Third, we analyzed whether the p120ctn-depleted mESCs could integrate in developing embryos and form chimeric animals. For this we used a diploid embryo complementation assay for both control and p120ctn-null mESCs. Control cells were aggregated with wild-type morulas and gave rise to multiple high-grade chimeras (Fig 3H). In contrast, p120ctn-null mESCs did never yield chimeric newborns, except in only one case where a very low-grade chimera was obtained (Fig 3I). Thus, although p120ctn-null mESCs can induce lineage commitment, their differentiation potential is obviously reduced *in vitro*. *In vivo*, the differentiation potential is dramatically reduced and mutant cells appear to be completely outcompeted by wild-type cells from the acceptor embryo.

### p120ctn is required for junctional maturation of primitive endoderm in EBs

**Fig 4:**
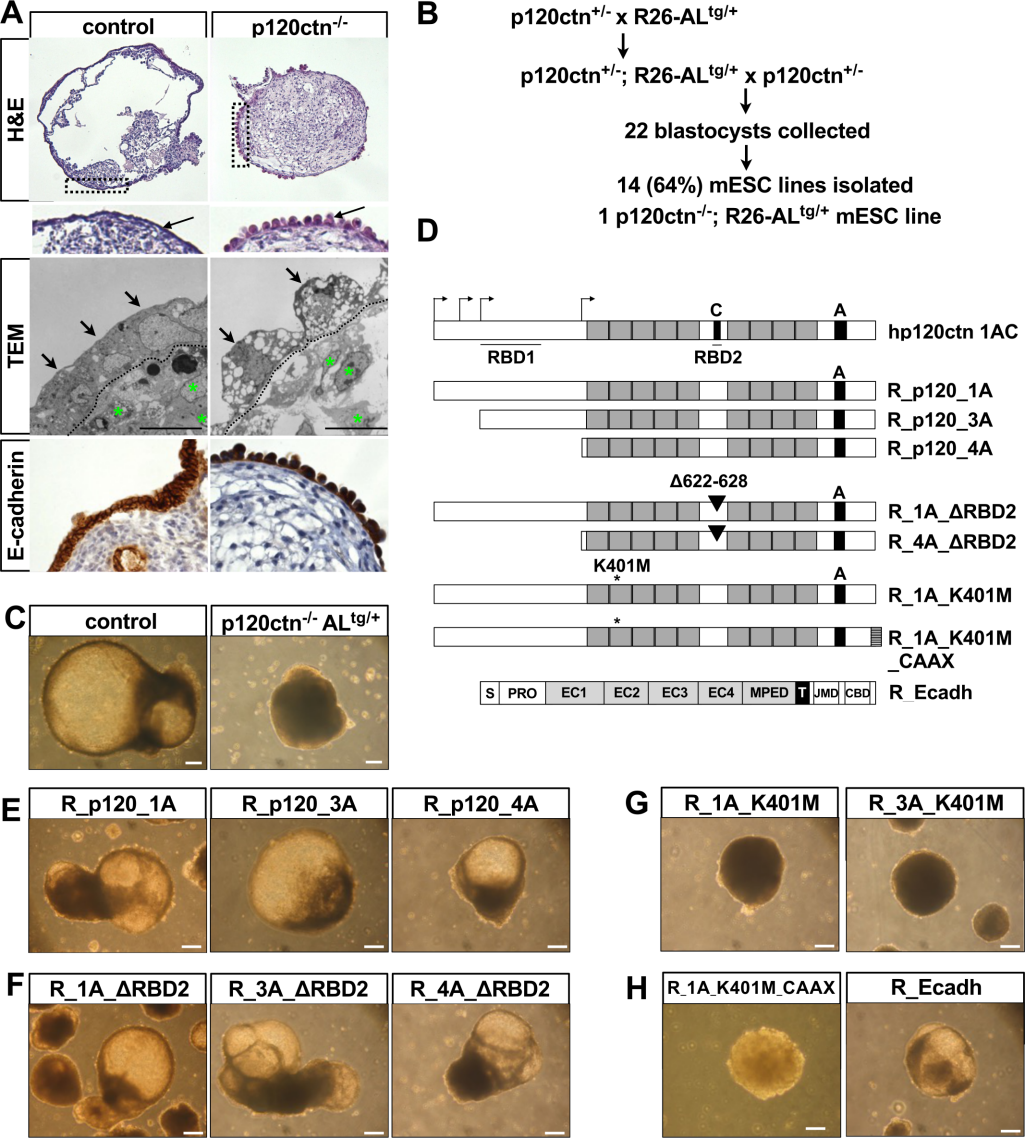
A structure-function based screening scrutinizes the p120ctn domains required for cystic EB formation. **(A)** Morphology (H&E-staining, top panels), ultrastructure (TEM, middle panels) and immunohistochemistry for E-cadherin (bottom panels) of control and p120ctn-null DIV30 EBs. Boxed area in H&E pictures is magnified to visualize the single-layered endodermal layer (arrow). The dashed line in the TEM micrographs separates the endodermal cells (arrows) from the inner EB cells (asterisk). Scale bars: 10 μm. **(B)** Breeding strategy and mESC isolation procedure to obtain p120ctn^-/-^ mESCs that harbor a recombination-mediated cassette exchange (RMCE)-compatible anti-Luc (AL) allele in the ROSA26 (R26) locus. **(C)** Micrographs showing DIV30 control and p120ctn-null EBs. Scale bars: 200 μm. **(D)** Schematic representation of coding potential of key rescue constructs that were introduced into the R26 locus of p120ctn^-/-^;AL^tg/+^ mESCs. See Table 1 for the complete list of rescue constructs. p120ctn isoforms contain a central armadillo domain consisting of nine armadillo repeats (grey boxes), one out of four alternative start codons (arrows), and possibly sequences encoded by alternatively used exons A and C (black boxes), and including or excluding two Rho GTPase binding domains (RBDs). p120ctn isoforms 1A, 3A and 4A use the first, third and fourth translation initiation site, respectively. The position of mutations of important AA and the artificial addition of a membrane-targeting motif (CAAX) are shown. E-cadherin (Ecadh) consists of a signal peptide (S), a pro-domain (PRO), four extracellular cadherin (EC) repeats, a membrane-proximal extracellular domain (MPED), a transmembrane domain (T), a juxtamembrane domain (JMD) and a β-catenin-binding domain (CBD). **(E-H)** Micrographs of DIV30 EBs after introduction of various rescue constructs by RMCE of mESCs, as indicated by the names on top of the images. Scale bars: 200 μm.

The outer cell layer of embryoid bodies forms an epithelium quite similar to the embryonic primitive endoderm, and has therefore been used as a model of hypoblast development [9, 35]. Histological analysis revealed that DIV30 EBs made with control mESCs formed a well polarized single layer of endodermal cells whereas this polarization was absent in p120ctn-depleted EBs (Fig 4A, top panels, arrows). Using transmission electron microscopy (TEM), we observed that the endoderm cells within control EBs had fully polarized matured cell–cell junctions including tight junctions (TJ), adherens junctions (AJ), desmosomes and gap junctions, whereas endodermal cells in p120ctn-null EBs had only minimal areas of cell–cell adhesion (Fig 4A, middle panels, arrows; S4 Fig).

The formation of an apical cell membrane with microvilli indicates well-established cell polarization and this was indeed observed on endodermal cells within the control EBs (S4A Fig). Remarkably, in p120ctn-null EBs, microvilli were observed throughout the cell surface and appeared even close to the few areas with minimal cell–cell adhesion (S4D and S4E Fig). We postulate that in p120ctn-null EBs, the limited adhesion sites, which lack occluding TJs, are not strong enough to support and maintain fluid-filled cystic EB structures.

Furthermore, also the cells that are enclosed within the endodermal layer of DIV30 p120ctn-null EBs showed much less cell–cell adhesion than control EBs (Fig 4A, middle panels, green asterisks). Collagen fiber expression was increased in p120ctn-null EBs (S4C Fig). These fibers provide physical support, allow the formation of three-dimensional structures such as EBs [36], and probably prevented p120ctn-null EBs from falling apart. We also tested whether the polarization defect seen in p120ctn-null EBs was caused by the E-cadherin levels being diminished as a result of p120ctn loss. Therefore, we stained EBs for E-cadherin and found similar staining in the endodermal layers of both control EBs and p120ctn-null EBs (Fig 4A, bottom panels). Overall our results indicate that p120ctn is essential for proper maturation of junctions and establishment of apical-basal polarity in the primitive endodermal layer of EBs and that this is required for the formation of cystic EBs.

### Performing p120ctn structure-function studies in p120ctn-null mESCs

Since p120ctn is a versatile multi-domain protein [16], we took a structure-function approach to pinpoint the molecular interactions that caused the defects in p120ctn-ablated mESCs and EBs. To this end, we generated recombinase-mediated cassette exchange (RMCE)-compatible p120ctn-null mESCs. This particular mESC line, named p120ctn^-/-^;AL^tg/+^ mESCs, was obtained by crossing heterozygous p120ctn-null mice with ROSA-AntiLuc (AL) mice (Fig 4B) [37]. The p120ctn^-/-^;AL^tg/+^ mESCs generated had a normal karyotype and recapitulated the phenotype that was previously observed in p120ctn-depleted EBs (Fig 4C). We used them for targeted introduction of various candidate rescue constructs via Flpe-mediated RMCE in the ROSA26 (R26) locus (S5A Fig).

The rescue constructs included a series of wild-type p120ctn isoforms, p120ctn mutants or downstream effectors. In total, we report here on 19 different rescue constructs, used for generation of 133 targeted mESC clones (Table 1). This occurred with very high targeting efficiency, often up to 100% (Table 1). Thus, this RMCE technology allowed us to generate in a semi high-throughput fashion a wide panel of mESC lines with various R26-based constructs, enabling *in vitro* structure-function studies in a p120ctn-null background.

**Table 1:** Targeted ESC lines generated by RMCE.

RMCE-targeted ESC lines	cDNAs	# of colonies picked	Targeting efficiency	EB morph-ology	Endoderm polarization (TEM)	Endodermal differentiation
**p120ctn isoforms**						
R_p120_1A	hp120ctn1A	6	100%	cystic	yes	yes
R_p120_3A	hp120ctn3A	18	89%	cystic		
R_p120_4A	hp120ctn4A	6	100%	cystic		
R_p120_1AC	hp120ctn1AC	6	67%	cystic		
R_p120_3AC	hp120ctn3AC	18	89%	cystic		
**p120ctn mutants**						
R_1A_ΔRBD2	hp120ctn1A_Δ622–628	6	100%	cystic		
R_3A_ΔRBD2	hp120ctn3A_Δ622–628	18	100%	cystic		
R_4A_ΔRBD2	hp120ctn4A_Δ622–628	6	100%	cystic		
R_1A_K401M	hp120ctn1A_K401M	6	83%	basic	no	no
R_3A_K401M	hp120ctn3A_K401M	6	100%	basic		
R_1A_K401M_CAAX	hp120ctn1A_K401M_CAAX	1	100%	basic	no	no
R_3A_K401M_CAAX	hp120ctn3A_K401M_CAAX	6	100%	basic		
**RhoGTPases**						
R_RhoA_DN	hRho DN (T19N)	4	100%	basic		
R_RhoA_CA	hRho CA (Q63L)	4	100%	basic		
R_Rac1_DN	hRac1 DN (N17L)	4	100%	basic		
R_Rac1_CA	hRac1 CA (G12V)	4	75%	basic		
R_Cdc42_DN	hCdc42 DN (T19N)	4	100%	basic		
R_Cdc42_CA	hCdc42 CA (Q61L)	4	75%	basic		
**Cadherins**						
R_Ecadh	hEcadherin	6	83%	cystic	yes	yes
**Total**		133	93%			

### All major wild-type p120ctn isoforms support cystic EB formation

**Fig 5:**
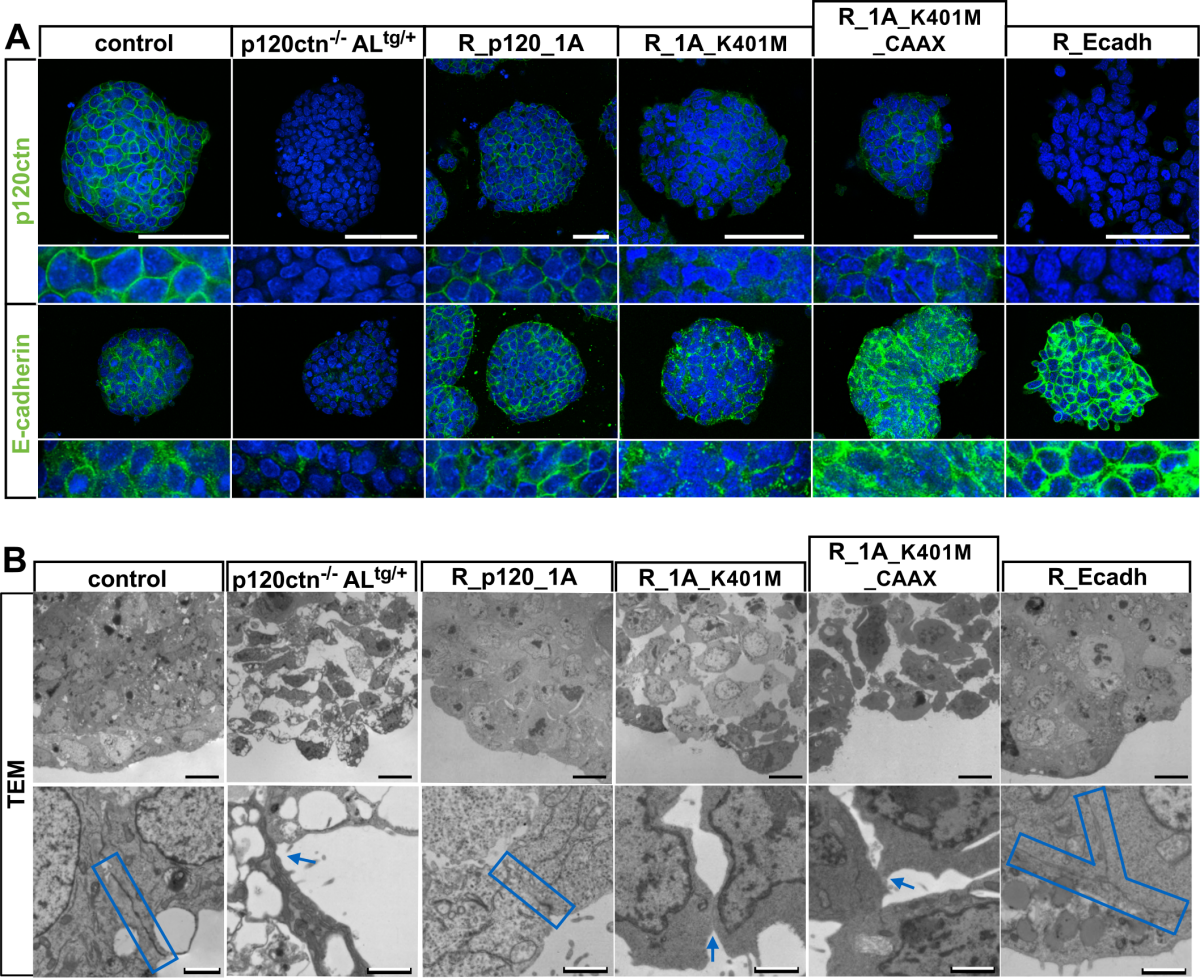
E-cadherin stabilization by p120ctn is required for proper polarization and cell–cell adhesion by EB cells. **(A)** Confocal pictures of p120ctn and E-cadherin stainings of control and p120ctn^-/-^;AL^tg/+^ mESCs, and of p120ctn^-/-^;AL^tg/+^ mESCs expressing various rescue constructs from the R26 promoter, as indicated by the names above each of the images. An 2.9-fold magnified image is shown below each picture. Scale bars: 25 μm. **(B)** TEM of DIV12 EBs, control or p120ctn^-/-^;AL^tg/+^ EBs, or p120ctn^-/-^;AL^tg/+^ EBs expressing various rescue constructs from the R26 promoter, as indicated. Blue boxes depict areas with mature junctions while blue arrows denote areas of minimal cell–cell adhesion. Black scale bars: 10 μm; white scale bars: 1 μm.

Multiple p120ctn isoforms [24] (Fig 4D) were investigated for their ability to rescue cystic EB formation. As proof of principle, RMCE-targeted mESCs expressing R26-driven wild-type p120ctn isoform 1A (designated R_p120ctn_1A) fully rescued E-cadherin levels at the plasma membrane of mESCs (Fig 5A), and formed cystic EBs (Fig 4E) as result of the reestablishment of mature adhesive contacts among outer endodermal cells (Fig 5B). The p120ctn levels in R_p120ctn_1A mESCs were about half of those observed in control mESCs (S5B Fig), but were sufficient to fully rescue the p120ctn null phenotype. In addition, we generated rescue lines, which expressed either p120ctn isoform 3A (R_p120_3A) or 4A (R_p120_4A) from the R26 locus. Both these introduced p120ctn isoforms were present at the cell surface and stabilized E-cadherin levels (S5C Fig), and hence rescued the defect in cystic EB formation (Fig 4E). This implies in particular that the N-terminal domain, as present in p120ctn isoforms 1A or 3A, is not required for p120ctn-dependent mESC differentiation and cystic EB formation.

### Binding and activation of Rho GTPases by p120ctn are not required for p120ctn-dependent cystic EB formation

As the results of the previous experiments suggest that the armadillo domain and the C-terminal region of p120ctn are sufficient for proper mESC differentiation, we studied the binding motifs for E-cadherin and RhoA, which are embedded in the armadillo domain. Two RhoA binding domains in p120ctn (RBD1 and RBD2, Fig 4D) are necessary for p120ctn-mediated regulation of RhoA activity. We generated mESCs with R26-based expression of p120ctn isoforms lacking either RBD2 (mutant 1AΔRBD2) or both RBDs (mutant 4AΔRBD2) (Fig 4D). We observed that the RBD2 sequence (AA 622–628) is modified naturally by the possible insertion of six AAs, which are encoded by the small alternatively spliced exon C [24]. We found that the inclusion of this six-AA insert blocks nuclear translocation and prevents dendritic branching upon overexpression (S6A–S6D Fig), a phenotype similar to that of the reported p120ctnΔ622–628 mutation [19]. When mESCs expressed in a p120ctn-deficient background either p120ctn isoforms with a Δ622–628 deletion (R_p120_1AΔRBD2, -3AΔRBD2 and -4AΔRBD2), or exon-C containing p120ctn constructs (R_p120_1AC and -3AC), they were all capable of forming cystic EBs (Fig 4F, S6E Fig). Hence, the interaction of p120ctn with RhoA is not essential for proper mESC differentiation or survival. In agreement with this, the subcellular localization of RhoA was not altered in p120ctn-depleted mESCs (S6F Fig).

However, p120ctn does modulate Rho GTPase activity [18]. Hence, we examined whether altered Rho GTPase activity upon p120ctn loss may cause the reduced endoderm polarity in EBs. For this, we generated p120ctn^-/-^ mESC rescue lines expressing either constitutively active (CA) or dominant-negative (DN) versions of each of the representative Rho GTPase family members, RhoA, Rac1 and Cdc42. In all these rescue attempts, the mutant mESCs failed to differentiate into cystic EBs (S6G Fig). Altogether, this implies that the endoderm polarization block in p120ctn-depleted mESCs occurs independently of p120ctn modulation of Rho GTPase activity levels.

### p120ctn binding to E-cadherin is required for endoderm polarization in EBs

**Fig 6:**
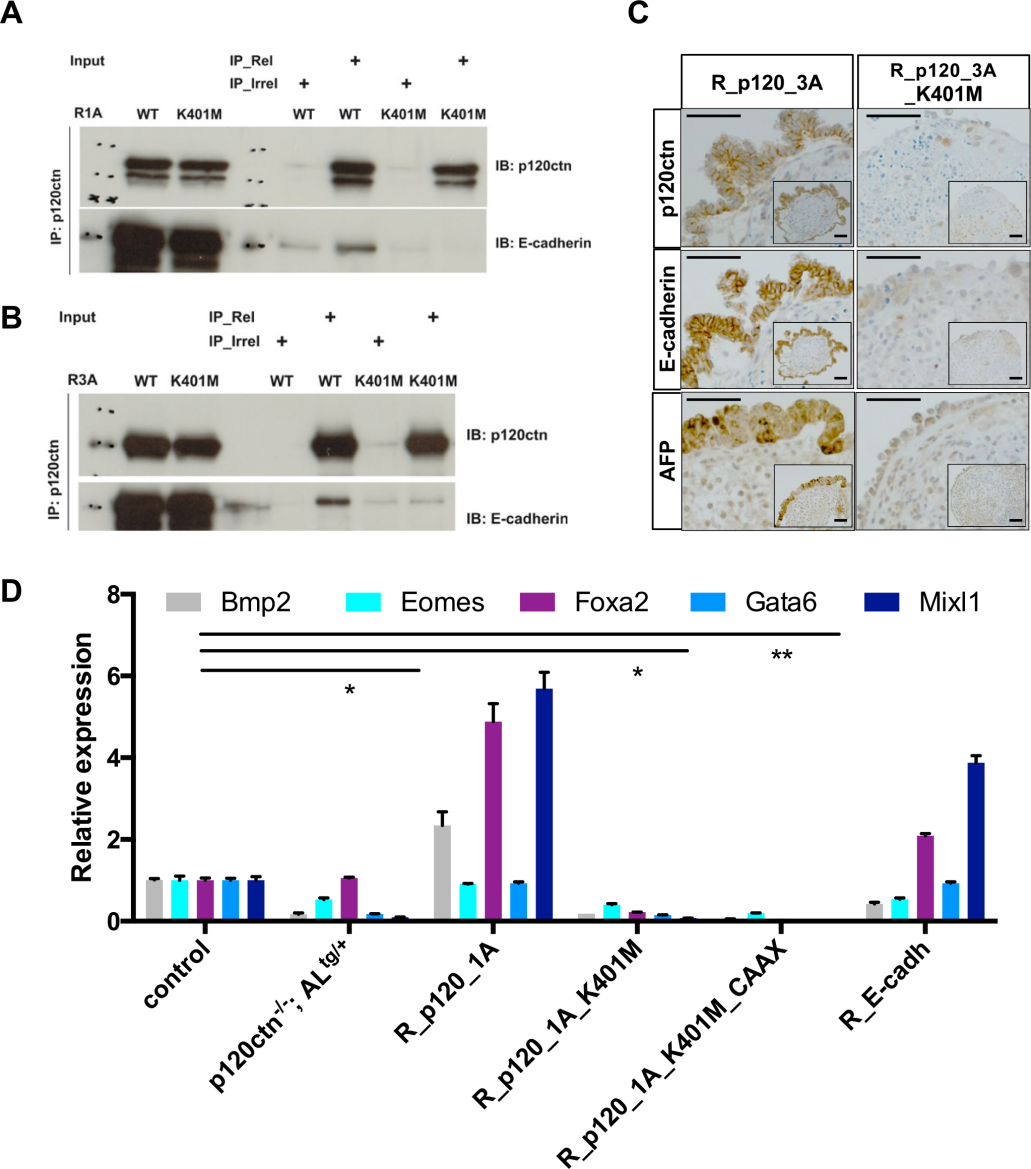
Proper expression of p120ctn and E-cadherin is required for endoderm differentiation from mESCs. **(A)** Immunoprecipitation (IP) experiments for p120ctn-null mESCs with R26-based expression of p120ctn isoform 1A (R1A WT) or its corresponding mutant (R1A K401M). **(B)** IP experiments for p120ctn-null mESCs with R26-based expression of p120ctn isoform 3A (R3A WT) or its corresponding mutant (R3A K401M). IPs were performed with an anti-p120ctn antibody (IP_Rel) or with an irrelevant anti-GFP antibody (IP_Irrel). Eluates immunoblotted (IB) with an anti-p120ctn antibody (top panels) showed that p120ctn was efficiently bound to the beads. Immunoblotting with an anti-E-cadherin antibody (bottom panels) confirmed the interaction of wild-type p120ctn with E-cadherin, whereas mutated K401M p120ctn was unable to bind E-cadherin. **(C)** Immunohistochemistry for p120ctn, E-cadherin and AFP on DIV30 p120ctn^-/-^ EBs expressing from the endogenous R26 promoter either p120ctn isoform 3A (R_p120_3A) or its E-cadherin-uncoupled K401M mutant form (R_p120_3A_K401M). Scale bars: 50 μm. **(D)** qRT-PCR analysis for endoderm-specific marker genes was performed using cDNAs originating from DIV12 control and p120ctn^-/-^;AL^tg/+^ EBs, and from p120ctn^-/-^;AL^tg/+^ EBs expressing various rescue constructs from the R26 promoter as indicated by the EB names. *Tbp* and *Rpl13a* were used for normalization. The error bars in the graphs represent the standard deviation of three technical replicates. * or ** denote comparisons that are significantly different. The *P* values (t test) from left to right are as follows: 0.016, 0.014 and 0.0018.

Next, we examined whether p120ctn-mediated stabilization of E-cadherin is required for proper mESC differentiation and EB formation. Previous structural analysis identified critical residues in p120ctn, such as K401, the mutation of which prevents the binding of p120ctn to E-cadherin [38]. We performed an R26-based rescue in p120ctn-null mESCs with such E-cadherin-uncoupled p120ctn mutants containing a single missense mutation (K401M, Fig 4D). The K401M mutant version of p120ctn isoforms 1A and 3A no longer bound to E-cadherin (Fig 6A and 6B). In contrast to the respective wild-type p120ctn isoforms, these mutant proteins resided not at the plasma membrane but in the cytoplasm (Fig 5A, S7A Fig). We then examined whether the dome-shaped morphology of mESC colonies was affected when p120ctn and E-cadherin were uncoupled. When cultured on MEFs the morphology of p120ctn-null mESC was normal. But when these cells were cultured feeder-free, the adhesion between mESCs was partially lost, along with the typical dome-shape (S7B Fig). Interestingly, when we tested their rescue, the p120ctn isoform 1A, but not the E-cadherin-uncoupled p120ctn_1A_K401M, restored this typical morphology in feeder-free conditions (S7B Fig).

Whereas mESCs that express p120ctn isoforms 1A or 3A readily formed cystic EBs in differentiation conditions (Fig 4E), their K401M mutant counterparts could not (Fig 4G); the latter formed almost exclusively basic EBs, and TEM analysis confirmed loss of endoderm polarization in such EBs (Fig 5B, arrow). These findings imply that the binding of p120ctn to E-cadherin is important for proper junction maturation within EBs.

To distinguish between p120ctn-dependent and E-cadherin-dependent molecular events, we created additional tools to manipulate the expression and the subcellular localization of either molecule. We observed in mESCs that p120ctn translocated from the plasma membrane to the cytoplasm when E-cadherin was lost (Fig 1B) or uncoupled (Fig 5A). To study whether the defects of p120ctn-deficient EBs were caused by sequestration of p120ctn away from the plasma membrane or were mainly due to reduced E-cadherin levels, we either forced the membrane relocalization of the E-cadherin-uncoupled p120ctn mutants or rescued the E-cadherin levels in p120ctn-null mESCs. To achieve membrane localization of the p120ctn K401M mutant, we fused the K-Ras membrane targeting motif (AAs CAAX) [39] to the carboxy-terminus of the p120ctn K401M mutant (designated as R_1A_K401M_CAAX), and confirmed its restoration of plasma membrane localization (Fig 5A). However, this relocalization could not restore the cell-cell adhesion defects (Fig 4H, Fig 5B). To test whether increased E-cadherin rescues differentiation defects caused by deletion of p120ctn, we generated p120ctn-null mESCs with supplemental R26-driven E-cadherin (R_Ecadh). The E-cadherin levels in such mESCs were obviously higher than in the parental p120ctn^-/-^;AL^tg/+^ mESCs (Fig 5A). This increase was sufficient for restoring the cell–cell adhesion (Fig 5B) and also rescued cystic EB formation (Fig 4H). Collectively, our data imply that p120ctn-mediated modulation of E-cadherin is required for establishing mature polarized junctions between endodermal cells, enabling the formation of cystic EBs.

### Proper cadherin levels are required for endoderm differentiation

We wondered whether p120ctn loss also influenced the commitment of such mESCs towards the endodermal lineage. The expression of endodermal markers was indeed reduced upon p120ctn loss (Fig 3G). Unlike p120ctn isoform 3A, the K401M p120ctn mutant failed to restore functional adhesion among endodermal cells in EBs, as judged by expression analysis of p120ctn, E-cadherin and AFP (Fig 6C).

To dissect the precise role of p120ctn during mESC-derived endodermal differentiation, we analyzed a panel of mESCs under EB DIV10 differentiation conditions and monitored several endoderm-related marker genes. In line with the aforementioned observations, p120ctn-null EBs failed to upregulate these markers with the apparent exception of Foxa2 (Fig 6D), demonstrating that p120ctn plays a key role in endoderm specification. Strikingly, this defective endoderm priming was convincingly rescued in cells with R26-driven wild-type p120ctn isoform 1A (Fig 6D), whereas, the E-cadherin-uncoupled p120ctn K401M mutant did not support endoderm differentiation; neither did membrane-targeted p120ctn K401M-CAAX (Fig 6D). Interestingly, ectopic expression of E-cadherin restored the endodermal markers. Of note, several endodermal genes were markedly higher in R_p120_1A compared to control EBs. Possibly the levels of p120ctn and its interaction with E-cadherin are regulated in a dynamic and temporal fashion during physiological endoderm specification. In contrast, the R26-driven rescuing provides a steady p120ctn expression level what potentially leads to enhanced endoderm signaling. This implies that a major role of p120ctn in endodermal differentiation is stabilization of cadherin levels at the plasma membrane.

## Discussion

We used mESC-derived EBs as a model for studying early embryonic epithelial differentiation. When cultured in suspension, mESCs self-aggregate and develop into a structure with a central cavity formed by apoptosis and surrounded by two epithelial layers, an outer polarized endoderm and an internal pseudostratified ectoderm, which are separated by a basement membrane [40]. The outer polar epithelium resembles the hypoblast and its subsequent visceral endoderm, while the internal columnar layer resembles the embryonic ectoderm or epiblast. Precursor cell populations of both cell types are initially distributed in the ICM in a ‘salt and pepper’ pattern [41], but later they are resolved into these two well-defined cell layers [42]. This cell sorting occurs in a cell autonomous fashion and does not require E-cadherin-based adhesion [43]. Our observations in p120ctn-null EBs confirm these reports. Although loss of p120ctn diminished E-cadherin levels, this cell sorting occurred normally. However, adhesion in both outer and internal cell layers was severely compromised in p120ctn-null EBs (Fig 4A). In particular, the external endoderm layer was morphologically distinct from that of control EBs. The latter displayed on their exterior surface a dense coat of microvilli, roughly 100 nm in diameter, and which is similar to this present on the surface of visceral endoderm cells in early-streak stage mouse embryos [44]. The fact that microvilli appear at the apical surface of endoderm cells is a strong hallmark of cellular polarity. This feature was strikingly lacking in p120ctn-null EBs, in which microvilli were either reduced or completely mislocalized. This implies that p120ctn is a crucial determinant of endodermal cell polarity.

Deregulation of cell polarity signaling may lead to change of cell fate. There are four modes of cell polarity: planar cell, apical-basal, front-rear, and mitotic spindle polarity. Apical-basal polarity is present in EBs and is involved in proper development [45]; its maintenance mainly depends on both cell polarity complexes and cell junctional complexes. Its formation starts with the establishment of various cell junctional complexes including lateral AJs, gap junctions, desmosomes and apical TJs. AJs and TJs can interact with cell polarity complexes. Protein kinase Cζ (PKCζ) is activated by Cdc42 and is associated with TJ assembly and apical polarization of epithelial cells. PKCζ translocates from the apical membrane to the cytoplasm upon conditional p120ctn loss in embryonic pancreas [46]. This p120ctn-dependent loss of apical polarization affects normal tubulogenesis in developing mouse pancreas and demonstrates the importance of p120ctn in the polarization of endoderm-derived organs *in vivo*. Possibly, p120ctn is an integral part of the apical-basal polarity complex, because it is dephosphorylated in response to activation of PKC family members [47]. Moreover, p120ctn has been identified as a key molecule in two spatially and functionally distinct junctional complexes: one involves PLEKHA7 and miRNA processing factors at the apical zonula adherens, and the other contains active Src and phosphorylated p120ctn at basolateral adhesion sites [48]. As p120ctn loss affects both types of junctional complexes, this will have severe consequences for both cell-cell adhesion and apical-basal polarity. Indeed, our results confirm that p120ctn is of crucial importance for TJ formation and apical-basal polarization in endodermal cells.

Interestingly, junctional complex formation and Cdc42 activation, which promote cellular polarity, are both p120ctn-regulated phenomena. AJs help to define the apical-basal axis in many epithelial cell systems and, by doing so, act as a reference point for the coordination of cell polarity across the epithelial sheet [49]. However, the establishment of apical polarity in primitive endoderm was reported to occur also in E-cadherin-null EBs [45]. Strikingly, we demonstrated that p120ctn-null EBs fail to form mature cell–cell contacts such as occluding TJs amongst endodermal cells, and as such are unable to establish or maintain an apical-basal architecture. TJs are present in the trophectoderm of blastocysts, seal the blastocoel and create a barrier that is almost impermeable to fluid [50]. Hence, p120ctn-null endodermal cells might fail to support cystic EB formation because they lack TJs. Consequently, cystic EB formation can be considered a useful indicator for well-polarized endoderm. Hence, we used this feature as an easy read-out for our rescue experiments (see below). On the other hand, p120ctn regulates Cdc42 [51, 52], which is a key Rho GTPase family member that coordinates reorganization of cytoskeletal systems along the apical-basal axis [53]. Furthermore, AJs and Cdc42 signaling are interconnected, as Cdc42 and Par proteins regulate the trafficking of AJ components, at least in *Drosophila* neuroectodem [54]. Since p120ctn ablation leads to near complete loss of cell-cell adhesion, it was difficult for us to distinguish between direct consequences of p120ctn loss and indirect effects due to loss of contact-dependent signaling. Therefore, we needed a way to identify the precise function or interactor of p120ctn that drives polarity of endodermal cells.

To this end, we performed structure-function analysis in p120ctn-null mESCs, using RMCE technology. Rescue cDNAs could be inserted into the R26 locus with unprecedented targeting efficiencies up to 100%, whereas targeting this locus by homologous recombination typically results in efficiencies of only about 20% [55]. This allowed us to comprehensively analyze the phenotypes observed not only in p120ctn-null mESCs/EBs, but also assess R26-driven rescue by wild-type p120ctn isoforms, p120ctn mutants and downstream effector proteins. There are three main p120ctn isoforms, namely isoform 1 (long isoform), isoform 3 (short isoform) and isoform 4 (shortest isoform). Long and short p120ctn isoforms display tissue-specific and cell-type specific expression, and a switch from short to long p120ctn isoforms is seen during epithelial-to-mesenchymal transition (EMT). Both isoforms behave differently in human cancers with respect to tumor proliferation, invasion and metastasis [26]. The aforementioned three p120ctn isoforms are produced from different start codons, which results in proteins with different N-terminal domain lengths. The N-terminal domain of p120ctn, contained in isoform 1 only, carries nearly all mapped p120ctn phosphorylation sites [25] and specifically interacts with kinases such as Fer, CK1 and GSK3β, but also with phosphatases SHP1 and RPTPμ (reviewed in [26]. It is well known that cadherin-based junctions are tethered to the actin cytoskeleton [56], but the N-terminal domain of p120ctn isoform 1 can also connect to microtubules, either directly by binding to kinesin [57, 58] or via PLEKHA7 [59]. Interestingly, R26-based expression of p120ctn isoform 4 in p120ctn-null mESCs is sufficient to rescue the cystic EB morphology, indicating that neither regulation by protein (de)phosphorylation nor the p120ctn-mediated linkage of cadherin-based junctions to microtubules is required for this process. This implies that probably the armadillo domain or possibly the C-terminal region of p120ctn plays an important role in endodermal polarization.

The armadillo domain enables interaction between p120ctn and E-cadherin, Kaiso, REST/CoREST and RhoGTPases [22, 60, 61]. Although RhoGTPases have been implicated in embryonic endoderm formation [62], we found that the p120ctn-dependent binding to and activation of Rho GTPases were not required for endoderm formation. For the transcriptional regulators Kaiso and REST/CoREST, their respective contributions to the p120ctn-null phenotypes are unclear. To discriminate between the adhesive and the transcriptional functions of p120ctn, we used the K401M mutant, previously documented to selectively uncouple E-cadherin from p120ctn, while leaving the binding to Kaiso intact [38] [63]. Failure of the K401M mutant to rescue the defects in p120ctn-null EBs strongly implicated E-cadherin, but not Kaiso, as crucial mediator of p120ctn-dependent endodermal specification. Next, we designed two experiments to exclude the possibility that the observed defects depended solely on p120ctn or that they were indirectly caused by a reduction in E-cadherin as a consequence of p120ctn loss. First, we used a CAAX-mediated membrane anchored E-cadherin-uncoupled p120ctn (K401M mutant). CAAX-mediated membrane targeting of p120ctn independently of E-cadherin ligation was previously shown to be sufficient to allow phosphorylation of p120ctn [64]. However, in our study such membrane targeting of the p120ctn K401M mutant failed to rescue endoderm polarization and differentiation. In contrast, R26-driven E-cadherin in p120ctn-null cells could fully rescue the defects seen upon loss of p120ctn. In line with this, Bedzhov and co-workers [65] elegantly showed that expression of E-cadherin, N-cadherin or chimeric E-/N-cadherin proteins from the endogenous E-cadherin promoter enables the formation of cystic EBs. In conclusion, we show for the first time that p120ctn acts as a key molecule that drives primitive endoderm specification and polarization in the early embryo, and that this function requires binding to and stabilization of junctional E-cadherin to do so.

## Materials and Methods

### Ethics statement

All experiments on mice were conducted according to institutional, national, and European animal regulations. Animal experiments were approved by the ethics committee of Ghent University.

### Mice and genotyping

Mice were housed in individually ventilated cages in a specific-pathogen-free animal facility. We used Ecadh^fl/fl^ mice, in which exons 4 to 15 of the *Cdh1* gene were floxed [66]. To generate p120ctn^+/-^ mice, floxed p120ctn^fl/fl^ mice [33] were crossed with Nestin-Cre mice [67], allowing Cre expression in all adult organs. The ROSA26-antiLuc reporter mouse was generated by homologous recombination in G4 mESCs [68] using the ROSA26-antiLuc construct [69]. B6/129 ROSA26-antiLuc F1-hybrid mice were subsequently outbred and maintained on a mixed B6/129/Swiss background. PCR primers are listed in Table 2.

**Table 2:** Primers for genotyping.

Allele	Primer name	Primer sequence	size (bp) of amplicon
p120ctn floxed	p120ctn_F	5’-TTGAACTCAGGACCGTCAGAGGAG-3'	wt: 450
	p120ctn_R1	5’-AAAGCAAGCCACCACCAACC-3'	floxed: 564
p120ctn null	p120ctn_F	5’-TTGAACTCAGGACCGTCAGAGGAG-3'	null: 550
	p120ctn_R2	5’-TCAGCACCCACACAAAGGTTG-3'	
E-cadherin floxed	Ecadh_F	5’-ACATGTTTGTATCGATCTCAG-3'	wt: 270
	Ecadh_R1	5’-CCATACACTGATAAT GTCAGA-3'	floxed: 330
E-cadherin null	Ecadh_F	5’-ACATGTTTGTATCGATCTCAG-3'	null: 320
	Ecadh_R2	5’-CCTGCCATGATTGTCA TGGAC-3'	
AntiLuc (AL)	ROSA26_F	5’-TAGGTAGGGGATCGGGACTCT-3'	1300
	ROSA26_R	5’-GCGAAGAGTTTGTCCTCAACC-3'	
RMCE targeted	RMCE_F	5’-AAAGTCGCTCTGAGTTGTTAT-3'	560
	RMCE_R	5’-GCGGCCTCGACTCTACGATA-3'	

### mESC culture, isolation and *in vitro* Cre excision

mESCs were cultured on gelatinized recipients containing mouse embryonic fibroblasts (MEFs, TgN (DR4)1 Jae strain) treated with mitomycin C (Sigma-Aldrich, St. Louis, MO). mESC isolation from blastocysts and subsequent culture was performed according to Pieters et al. [28]. mESCs were grown in SR-mESC medium alone, or in SR-mESCs medium containing 2 μM pluripotin (Cayman Chemical, Ann Arbor, MI) or supplemented with 2i (1 μM Erk inhibitor PD0325901 and 3 μM Gsk3 inhibitor CHIR99021). An overview of all mESC derivations is shown in Table 3. Floxed p120ctn and E-cadherin alleles were Cre excised *in vitro*, each time in two independent mESC lines by electroporation with 5 μg pCAG-NLS-Cre-IRES-Puro-pA. After 24 h, cells were subjected to puromycin selection (1.25 μg/ml; Sigma) for 4 days. For each mESC line, 12 puromycin-resistant colonies were picked, expanded and screened by PCR (Table 2), and finally immunostaining. All mESCs lines that were used had a normal karyotype (S2A and S2B Fig).

**Table 3:** The efficiency of derivation of mESC lines from blastocysts.

Mating	Compound added		# of blast-ocysts	primary outgrowths	# of mESC lines derived (efficiency)
E-cadherin^fl/fl^ intercross	pluripotin	15		15 (100%)	13 (87%)
p120ctn^fl/fl^ intercross	pluripotin	6		6 (100%)	6 (100%)
p120ctn^+/-^ intercross	pluripotin	10		10 (100%)	10 (100%)
p120ctn^+/-^;AL ^tg/+^ x p120ctn^+/-^	2i*	22		22 (100%)	14 (64%)

* ES cells were isolated according to Pieters et al., 2012a [16] but pluripotin was replaced by two inhibitors (2i), respectively, PD98059 blocking Erk and CHIR99021 blocking Gsk3.

### Plasmids

pENTR1A-hp120ctn 1A, 1AC, 1AΔ622–628, 3A, 3AC, 1AΔ622–628, 4A and 4AΔ622–628 were generated by ligation of p120ctn cDNA fragments, obtained by *Eco*RI and *Oli*I digestion from various eukaryotic expression vectors [70], into pENTR1A vectors that were digested with *Eco*RI and *Eco*RV. hp120ctn Δ622–628 mutants were created with a QuikChange II Site-Directed Mutagenesis Kit (Stratagene). p120ctn K401M mutants and p120ctn K401M-CAAX mutants were generated by cloning synthetic p120ctn DNA fragments (Genescript) into pENTR1A-hp120ctn 1A and 3A vectors. The pENTR3C-hEcadh-withSTOP plasmid with a native stop codon was obtained by using QuikChange (Agilent Technologies) on pENTR3C-hEcadh-NoSTOP (kind gift from Philippe De Groote). pENTR vectors containing constitutively active or dominant-negative Rho GTPases were a kind gift from Prof. Christophe Stove (Ghent University).

### RMCE targeting

RMCE-compatible p120ctn-null (p120ctn^-/-^;AL^tg/+^) mESCs were generated by crossing p120ctn^+/-^ mice with AL^tg/+^ mice [37] (Fig 4B). For the trap-coupled RMCE experiments, 50% confluent p120ctn^-/-^;AL^tg/+^ mESCs were cotransfected with the pRMCE-DV1 vector, from which the floxed stop cassette was removed by Cre [71], and a FlpE-expressing plasmid (pCAGGS-FlpE-IRES-puromycin-pA) [72] in a 1:1 ratio using Lipofectamin 2000 reagent (Invitrogen). G418 selection (200 μg/ml) was started 48 h after transfection. After 7 to 10 days, individual G418-resistant RMCE-targeted ES colonies were observed and were further expanded. For each targeting, four to six colonies were picked and validated by PCR (Table 2), followed by immunostaining. cDNAs of interest were cloned into the Gateway-compatible and Cre-excised pRMCE-DV1 vector using Gateway LR reactions as previously described [55]. Table 1 contains an overview of all RMCE targeted rescue lines made.

### EB formation and mESC differentiation

The procedure for generating EBs was similar to that described before [28]. For marker analysis, EBs were allowed to form for 10 d in bacterial grade Petri dishes. The EBs were then plated onto gelatinized culture dishes and allowed to attach and spread out for 10 days in differentiation medium. For long-term cultures, EBs were refreshed three times weekly. After 30 days, the EBs were trypsinized and then plated in 6-well plates at a density of 1 x 10^6^ cells/well, followed by culture for 4 days in SR-mESC medium and staining for alkaline phosphatase (AP) using the Leukocyte Alkaline Phosphatase Kit (Sigma).

### Karyotyping mESCs

The normal mouse mitotic karyotype consists of 40 acrocentric chromosomes. This was checked in our mESC lines by routine procedures [28]. Chromosome spreads were stained with DAPI and 20 individual chromosome sets were counted. An mESC line is considered normal if 70% or more of its spreads contain 40 chromosomes.

### Teratoma formation

Control and p120ctn-null mESCs were trypsinized and 1.5 x 10^6^ cells were injected subcutaneously into the flank of immunocompromised nude mice. Growing teratomas were isolated 5 to 8 weeks later and processed for histological analysis.

### Diploid embryo aggregation

Chimeras were generated by diploid embryo aggregation. Briefly, E2.5 embryos were collected from superovulating Swiss female mice and the zona pellucida of embryos was removed by treatment with acid Tyrode's solution (Sigma). mESC colonies from control and p120ctn-null mESCs were briefly treated with 0.25% trypsin-EDTA (Invitrogen) to form loosely connected clumps of 7 to 10 cells. Each zona-pellucida free embryo was aggregated with a clump of 7 to 10 cells using depression wells made with an aggregation needle (BLS Ltd, Hungary) in a 35-mm plastic dish (VWR International). Aggregates were cultured overnight in microdrops of KSOM with amino acids (Biognost) under mineral oil (Sigma) at 37°C in 95% air and 5% CO_2_. The next day, blastocysts were transferred into the uteri of 2.5-dpc pseudopregnant Swiss females previously mated with vasectomized males. Chimeras were identified at birth by the presence of black eyes and later by agouti coat pigmentation.

### Transmission electron microscopy (TEM)

EBs were fixed in 4% paraformaldehyde and 2.5% glutaraldehyde in 0.1 M sodium cacodylate buffer, pH 7.2 for 4 hours at room temperature followed by fixation overnight at 4°C. After washing in buffer, they were post fixed in 1% OsO_4_ with 1.5% K_3_Fe(CN)_6_ in 0.1 M sodium cacodylate buffer at room temperature for 1 hour. After washing in double distilled H_2_O, cells were subsequently dehydrated through a graded ethanol series, including a bulk staining with 1% uranyl acetate at the 50% ethanol step followed by embedding in Spurr’s resin. Ultrathin sections of a gold interference color were cut using an ultra-microtome (Leica EM UC6), followed by a post-staining in a Leica EM AC20 for 40 min in uranyl acetate at 20°C and for 10 min in lead stain at 20°C. Sections were collected on Formvar-coated copper slot grids and were viewed with a JEM 1010 transmission electron microscope (JEOL, Tokyo, Japan) operating at 60 kV using Image Plate Technology from Ditabis (Pforzheim, Germany).

### Immunostaining and immunohistochemistry

The staining procedure for mESCs, plated EB cells, and preimplantation embryos involved fixation with 4% paraformaldehyde, permeabilization with 0.2% Triton X-100 and incubation at room temperature for 2 h (for differentiated cells) or overnight (for mESCs) with primary antibody (Table 4), followed by incubation with secondary antibody (Molecular Probes, Eugene, OR) for 1 h (differentiated cells) or overnight (for mESCs). Confocal microscopy was performed using a Leica TCS SP5 confocal scan head attached to a Leica DM IRE2 inverted microscope. Image analysis was performed in Volocity 5.5 (Perkin Elmer). EBs and embryos (7.5 to 9.5 dpc) were fixed in 4% paraformaldehyde, embedded in paraffin and 5-μm sections were cut for staining. For immunohistochemistry, tissue sections were deparaffinized and rehydrated. Antigen retrieval was performed by heating the sections in 10 mM sodium citrate buffer (pH 6.0) in an electric pressure cooker, after which the slides were permeabilized with 0.05% Tween 20 in PBS. Blocking of endogenous peroxidase occurred in 3% H_2_O_2_ in methanol. Sections were then treated with 1% goat serum/1% BSA in PBS, followed by incubation with primary antibodies (Table 4) overnight at 4°C. Biotin-conjugated secondary antibodies (Dako, Heverlee, Belgium) were detected by the avidin-biotin complex (Vector Laboratories, Burlingame, CA, USA) and developed with diaminobenzidine (Dako).

**Table 4:** Primary antibodies.

Antibody raised against	Species	Dilution	Company
p120ctn (pp120)	Mouse monoclonal	1/500	BD Transduction Laboratories
p120ctn (pAb exC)	Rabbit polyclonal	1/50	Made in house
β-catenin	Rabbit polyclonal	1/2000	Sigma
α-catenin	Rabbit polyclonal	1/1000	Sigma
E-cadherin (DECMA-1)	Rat polyclonal	1/100	Sigma
E-cadherin (BD)	Mouse monoclonal	1/300	BD Transduction Laboratories
Oct4 (C-10)	Mouse monoclonal	1/200	Santa Cruz Biotechnology
Nestin	Mouse monoclonal	1/1000	Becton Dickinson
anti-βIII tubulin	Rabbit polyclonal	1/3000	BD Pharmingen
α-Fetoprotein 1 (AFP)	Rabbit polyclonal	1/500	Dako
CD45	Rat monoclonal	1/20	BD Pharmingen
Smooth muscle actin (SMA)	Mouse monoclonal	1/100	Sigma
α-Dystrobrevin (H-300)	Rabbit polyclonal	1/200	Santa Cruz
RhoA	Rabbit polyclonal	1/100	Sigma
PKCζ	Rabbit polyclonal	1/200	Santa Cruz

### qRT-PCR

Total RNA was isolated using RNeasy Plus Mini Kit (QIAGEN). cDNA was synthesized using the First Strand cDNA Synthesis Kit (Roche) with oligo(dT) primer starting from equal amounts of RNA as measured by a NanoDrop spectrophotometer (Thermo Scientific). qRT-PCR was performed using the LightCycler 480 SYBR Green I Master (Roche) and monitored on a LichtCycler 480 system (Roche). Gene expression was standardized against reference genes *Hmbs*, *Actb* and *Gapdh*. All primers are listed in Table 5.

**Table 5:** qRT-PCR primers.

Gene name	Forward (F) and reverse (R) primers
*Oct4*	F: 5’‐ACATCGCCAATCAGCTTGG‐3’
	R: 5’‐AGAACCATACTCGAACCACATCC‐3’
*Sox2*	F: 5’‐ACAGATGCAACCGATGCACC‐3’
	R: 5’‐TGGAGTTGTACTGCAGGGCG‐3’
*Klf4*	F: 5’‐GCACACCTGCGAACTCACAC‐3’
	R: 5’‐CCGTCCCAGTCACAGTGGTAA‐3’
*Nanog*	F: 5’‐CCACAGTTTGCCTAGTTCTGAGGAAGCATC‐3’
	R: 5’‐TACTCCACTGGTGCTGAGCCCTTCTGAATC‐3’
*ERas*	F: 5’‐ACTGCCCCTCATCAGACTGCTACT‐3’
	R: 5’‐CACTGCCTTGTACTCGGGTAGCTG‐ 3’
*Cripto*	F: 5’‐ATGGACGCAACTGTGAACATGATGTTCGCA‐3’
	R: 5’‐CTTTGAGGTCCTGGTCCATCACGTGACCAT‐ 3’
*Mixl1*	F: 5’‐ CTACCCGAGTCCAGGATCCA‐3’
	R: 5’‐ ACTCCCCGCCTTGAGGATAA‐3’
*Eomes*	F: 5’‐ GGCCTACCAAAACACGGATATC‐3’
	R: 5’‐ TTTCTGAAGCCGTGTACATGGA‐3’
*Foxa2*	F: 5’‐ CGAGTTAAAGTATGCTGGGAG‐3’
	R: 5’‐ TATGTGTTCATGCCATTCATCC‐3’
*Bmp2*	F: 5’‐GCTCCACAAACGAGAAAAGC‐3’
	R: 5’‐AGCAAGGGGAAAAGGACACT‐3’
*Gata6*	F: 5’‐GAACGTACCACCACCACCAT‐3’
	R: 5’‐CCATGTAGGGCGAGTAGGTC‐3’
*Hmbs*	F: 5’‐TCGGGGAAACCTCAACACC‐3’
	R: 5’‐CCTGGCCCACAGCATACAT‐3’
*β‐actin*	F: 5’‐ATGGTGACGTTGACATCCGTA‐3’
	R: 5’‐ATGGTGACGTTGACATCCGTA‐3’
*Gapdh*	F: 5’‐AGGTTGTCTCCTGCGACTTCA‐3’
	R: 5’‐GGTGGTCCAGGGTTTCTTACTC‐3’

### Nuclear translocation and branching assays

The mammalian expression vector pHM829 was designed for expression of proteins of interest fused to β-galactosidase (β-gal) at its N-terminus and to green fluorescent protein (GFP) at its C-terminus [73]. pHM829 vectors containing either the second NLS of p120ctn (NLS) or a mutated NLS (NLSmut) have been described [21]. To create a pHM829 construct containing the second p120ctn NLS interrupted by exon C-encoded amino acids, two complimentary oligonucleotides were designed to incorporate a 5′ *SacII* site and a 3ʹ *XbaI* site (underlined) flanking the NLS sequence, which is interrupted by p120ctn exon C (italics): 5’- CCGCGGAAGAAGGGCAAAG*ATGAGTGGTTCTCCAGAG*GGAAAAAGCCTTCTAGA-3’; 3’-GGCGCCTTCTTCCCGTTTC*TACTCACCAAGAGGTCTC*CCTTTTTCGGAAGATCT-5’. The oligonucleotides were annealed, digested with *SacII* and *XbaI*, and ligated in pHM829 pre-digested with the same enzymes.

HeLa cells were grown at 37°C under 5% CO_2_ in DMEM supplemented with 10% fetal bovine serum, 4 mM L-glutamine, penicillin (100 U/ml) and streptomycin (100 mg/ml). Cells were transfected by using Fugene reagent (Roche Applied Science). For each construct tested in the branching assay, at least 100 transiently transfected HeLa cells were scored for normal or branched cellular phenotype.

### Production and purification of a polyclonal antibody against p120ctn isoform C

An antibody specific for p120ctn isoform C (pAb exC) was generated in rabbits against a peptide containing the six AA encoded by alternative exon C (GKDEWFSRGKGAC) and fused to keyhole limpet hemocyanin (KLH, Sigma). This serum was characterized and validated by immunofluorescence of cells transiently transfected with either pEFBOS hp120ctn 3A or 3AC (S8A Fig), and by western blot analysis of mouse brain and gastrulating embryos (S8B Fig). The most specific serum was affinity purified on peptide-coupled columns prepared using the SulfoLink kit (Pierce Biotechnology). Antibody-containing fractions were identified by ELISA.

### Co-immunoprecipitation

mESCs were lysed in buffer containing 150 mM NaCl, 2 mM EDTA, 25 mM Tris-HCl, pH 8 and 0.5% Triton-X-100 supplemented with Protease Inhibitor Cocktail (Roche). The insoluble fraction was removed by centrifugation and the Bio-Rad DC assay kit was used for protein measurement of the lysate. 800 μg of protein was incubated for 2 h at 4°C with 5 μg of antibody, either mouse anti-p120ctn (BD Transduction) or, as an irrelevant antibody control, mouse anti-GFP (Invitrogen). After antibody incubation, 50 μl of protein-G Dynabeads (Invitrogen), previously washed in lysis buffer, were added and incubation was continued for 1 h at 4°C. The bead–protein complexes were washed 4 times with lysis buffer and a magnetic rack was used to collect the beads. Proteins were eluted from the beads by boiling the samples in SDS-loading buffer.

## Supporting Information

S1 Fig Characterization of wild-type and p120ctn-null blastocysts. **(A)** Confocal fluorescent images of a wild-type blastocyst stained for p120ctn and the apical marker PKCζ. p120ctn is expressed basolaterally. Scale bar: 25 μm. **(B)** Maternal p120ctn allows basal E-cadherin stabilization on the membranes of p120ctn-deficient blastocysts. Three-dimensional (3D) reconstruction using consecutive confocal sections of the blastocysts shown in Fig 2E. The limited amount of maternal p120ctn in p120ctn-deficient embryos (p120ctn^-/-^, arrowheads) probably allows the stabilization of basal E-cadherin levels (arrowheads) on the membranes of blastocysts. This is thought to be sufficient for normal compaction and blastocyst formation. Scale bar: 25 μm.
(PDF)

S2 Fig Characterization of p120ctn-null mESCs. **(A)** Chromosomal analysis of control and p120ctn-null mESCs. Fluorescent images of DAPI-stained mitotic spreads with 40 acrocentric chromosomes from control mESC lines (p120ctn^fl/fl^ (fl2, f21), p120ctn^+/+^ (+/+9), p120ctn^+/-^ (+/-1)) and p120ctn-null mESCs (-/-7, -/-12, -/-3, -/-8). **(B)** Graph depicts the percentage of mitotic spreads that contain 39, 40, 41, 42 and 43 or more chromosomes. An mESC line is considered normal if 70% or more of its spreads contain 40 chromosomes. The names of mESC lines analyzed are at the bottom. **(C)** Confocal fluorescent images of control and p120ctn-null mESCs stained for α-catenin and β-catenin. The boxed areas were further magnified 3.6-fold. Scale bars: 25 μm. **(D, E)** qRT-PCR analysis for expression of various stemness genes, in **(D)** control and p120ctn-null mESCs, and in **(E)** their corresponding EBs after 30 days of culture (DIV30). *Actb*, *Gapdh* and *Hmbs* were used as reference genes. The error bars in the graphs represent the standard error of the mean of two independent control or p120ctn-null cell lines.
(PDF)

S3 Fig Teratoma formation. p120ctn loss does not abrogate germ layer development. Histological analysis of H&E stained sections of teratomas from control (p120ctn^+/+^) and p120ctn-depleted (p120ctn^-/-^) mESCs. Scale bars: 100 μm.
(PDF)

S4 Fig Transmission electron microscopy. TEM analysis of DIV12 control EBs **(A, B)** and p120ctn-null EBs **(C-E)**. The red dashed box in **(D)** is enlarged in **(E)** and shows a region of minimal endodermal cell-cell adhesion (blue arrows) showing non-polarized microvilli (black arrows). Scale bars: 2 μm. AJ, adherens junction; DS, desmosome; TJ, tight junction.
(PDF)

S5 Fig RMCE targeting in p120ctn-null mESCs. **(A)** Scheme depicting our mouse breeding protocol, followed by isolation of the RMCE-compatible p120ctn^-/-^;AL^tg/+^ mESCs, and insertion of various rescue cDNAs in the ROSA26 locus by RMCE. By Gateway cloning we inserted a set of candidate rescue cDNAs (listed in Table 1) into an RMCE-compatible destination vector, called pRMCE-DV1, which also harbors two heterospecific Frt sites, which do not cross-react with each other (depicted by white and red triangles), followed by a PGK promoter and the start codon of the NeoR gene [71]. We co-transfected p120ctn^-/-^;AL^tg/+^ mESCs with the different pRMCE-DV1 plasmids and with a Flpe expression plasmid. Flpe-mediated cassette exchange inserted the gene of interest (GOI) into the ROSA26 locus and in addition restored neomycin-resistance. In these targeted mESCs, both the GOI and NeoR genes are driven by the endogenous R26 promoter. A fluorescent image of a DAPI-stained mitotic spread with 40 acrocentric chromosomes from p120ctn^-/-^;AL^tg/+^ mESCs is shown at the bottom. **(B)** Graph depicting p120ctn levels in control and p120ctn-null mESC, and in p120ctn-null mESC with R26-driven expression of p120ctn isoform 1A (R_p120_1A) or of its K401M mutant (R_1A_K401M). Z-stacks, optimized according to the Nyquist sampling theorem, were acquired on the SP5 Leica confocal microscope. A fixed intensity threshold was set on the Alexa 488 signal representing p120ctn staining. Within this threshold, the total amount of voxels for each mESC colony was counted and normalized against its total nuclear volume. At least 10 reconstructed colonies were analyzed for each mESC line. **(C)** Confocal fluorescent images of p120ctn-null mESCs with R26-driven expression of p120ctn isoform 3A (R_p120_3A) or 4A (R_p120_4A) stained for p120ctn or E-cadherin expression. A threefold magnified image is shown below each picture. Scale bars: 50 μm.
(PDF)

S6 Fig RhoA binding to p120ctn and RhoGTPase modulation are dispensable for cystic EB formation. Amino acids encoded by p120ctn exon-C inhibit nuclear translocation and dendritic-like branching. **(A)** Nuclear translocation assay using fusion proteins composed of an N-terminal β-galactosidase (β-gal) part and a C-terminal GFP. Between the β-gal and the GFP parts we cloned the NLS of p120ctn (NLS, AA622-628), a mutated version of it (NLSmut), or the NLS interrupted by amino acids encoded by exon-C (NLSexon-C). These constructs were expressed in HeLa cells. Confocal fluorescence analysis showed that both NLSmut and NLSexon-C prevented the nuclear GFP expression seen with the NLS construct. The exon-C encoded AA expressed by the NLSexon-C construct were also specifically detected by an in-house made polyclonal antibody (pAb exC, bottom panel). **(B, C)** Branching assay in HeLa cells transiently transfected with plasmids expressing either **(B)** p120ctn isoform 1A and related proteins, or **(C)** isoform 3A and related proteins. The related proteins were p120ctn isoform C variants (p120ctn isoforms 1AC and 3AC), or mutants lacking amino acids 622–628 (p120ctn isoforms 1AΔ and 3AΔ). Confocal images were made after immunostaining for all p120ctn isoforms (pp120, red), either combined or not combined with specific staining for p120ctn isoform C (pAb exC, green). **(D)** Graph showing the percentage of branched versus normal cellular phenotypes upon overexpression of different p120ctn isoforms in HeLa cells, as illustrated in panels **(B)** and **(C)**. **(E)** Micrographs of DIV30 p120ctn-null EBs with R26-driven expression of p120ctn isoform 1AC (R_p120_1AC) or 3AC (R_p120_3AC). Two independently rescued mESC lines were analyzed in each setup. Scale bars: 200 μm. **(F)** Confocal fluorescent pictures of control and p120ctn-null mESCs stained for p120ctn (green) and RhoA (red). A 3.3-fold magnification is shown below each picture. Scale bars: 15 μm. **(G)** Micrographs of DIV30 p120ctn-null EBs with R26-driven expression of dominant-negative (DN) or constitutively active (CA) Rho GTPases, as indicated. Two independently rescued mESC lines were analyzed in each setup. Scale bars: 200 μm.
(PDF)

S7 Fig Assays with E-cadherin-uncoupled p120ctn mutants. **(A)** Confocal fluorescent pictures of R_p120_3A mESCs, and of mESCs expressing the K401M mutant of p120ctn isoform 3, after immunostaining for p120ctn and E-cadherin. Scale bars: 25 μm. **(B)** Micrographs depicting the morphology of different mESC cultures, as indicated. Scale bars: 100 μm
(PDF)

S8 Fig Characterization of pAb ExC antibody. **(A)** Immunostaining to characterize an antibody specific for isoform C of p120ctn (pAb ExC). This antibody recognizes in transiently transfected MCF7 cells human p120ctn isoform 3AC, but not human isoform 3A lacking exon-C encoded amino acids. Monoclonal antibody pp120 recognizes all p120 isoforms. **(B)** Western blot analysis of two wild-type gastrulating mouse embryos (E7.5) in which all endogenous p120ctn isoforms were detected with pp120 antibody and endogenous p120ctn isoforms C were detected with pAb ExC. Mouse brain was taken as a positive control because it is relatively rich in isoforms C of p120ctn.
(PDF)
