# Supplementary figures and images for: p120 Catenin-Mediated Stabilization of E-Cadherin Is Essential for Primitive Endoderm Specification
Source: PLoS Genet. 2016 Aug 24;12(8):e1006243. doi: 10.1371/journal.pgen.1006243 (PMC4996431; doi:10.1371/journal.pgen.1006243)

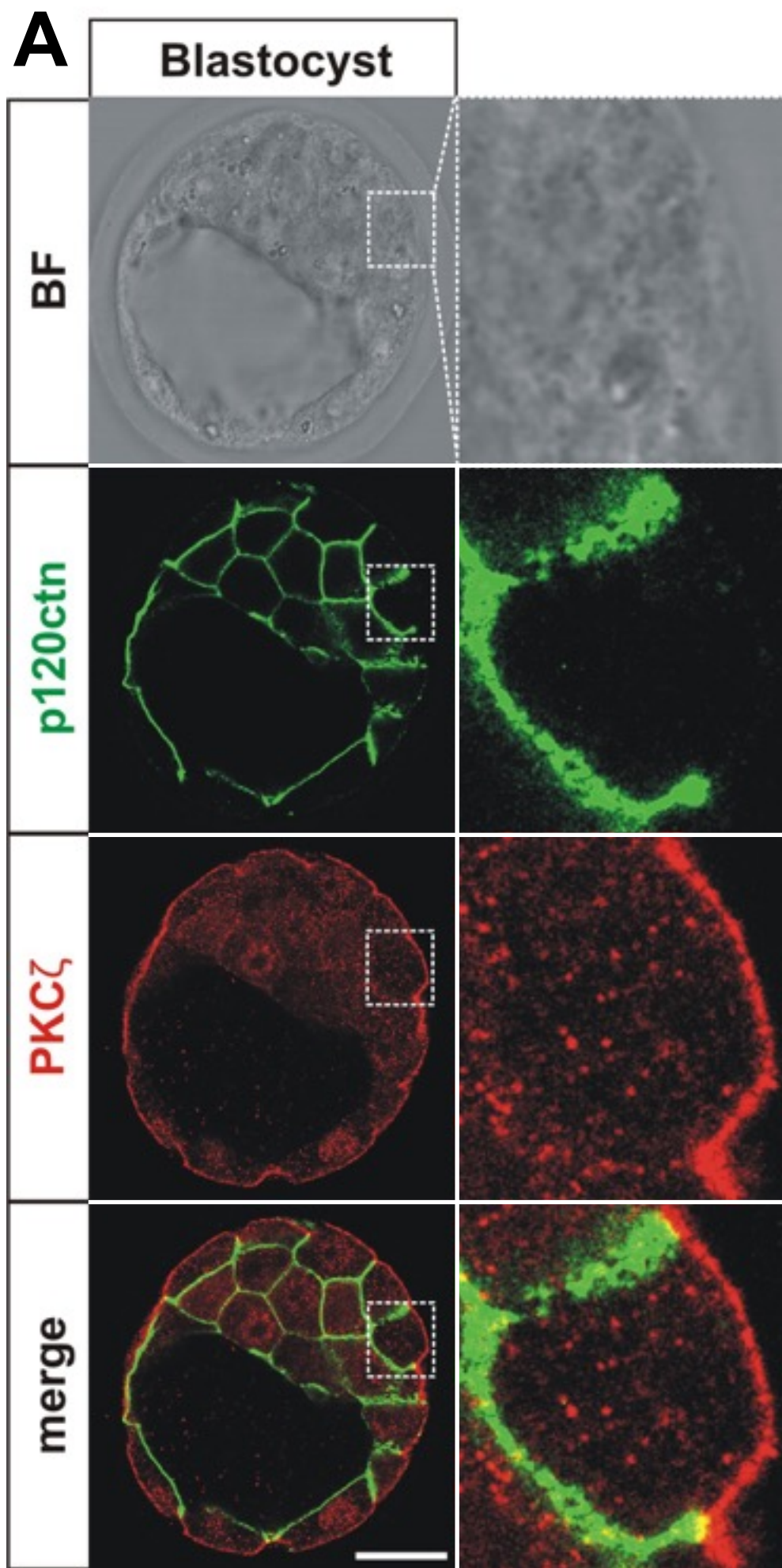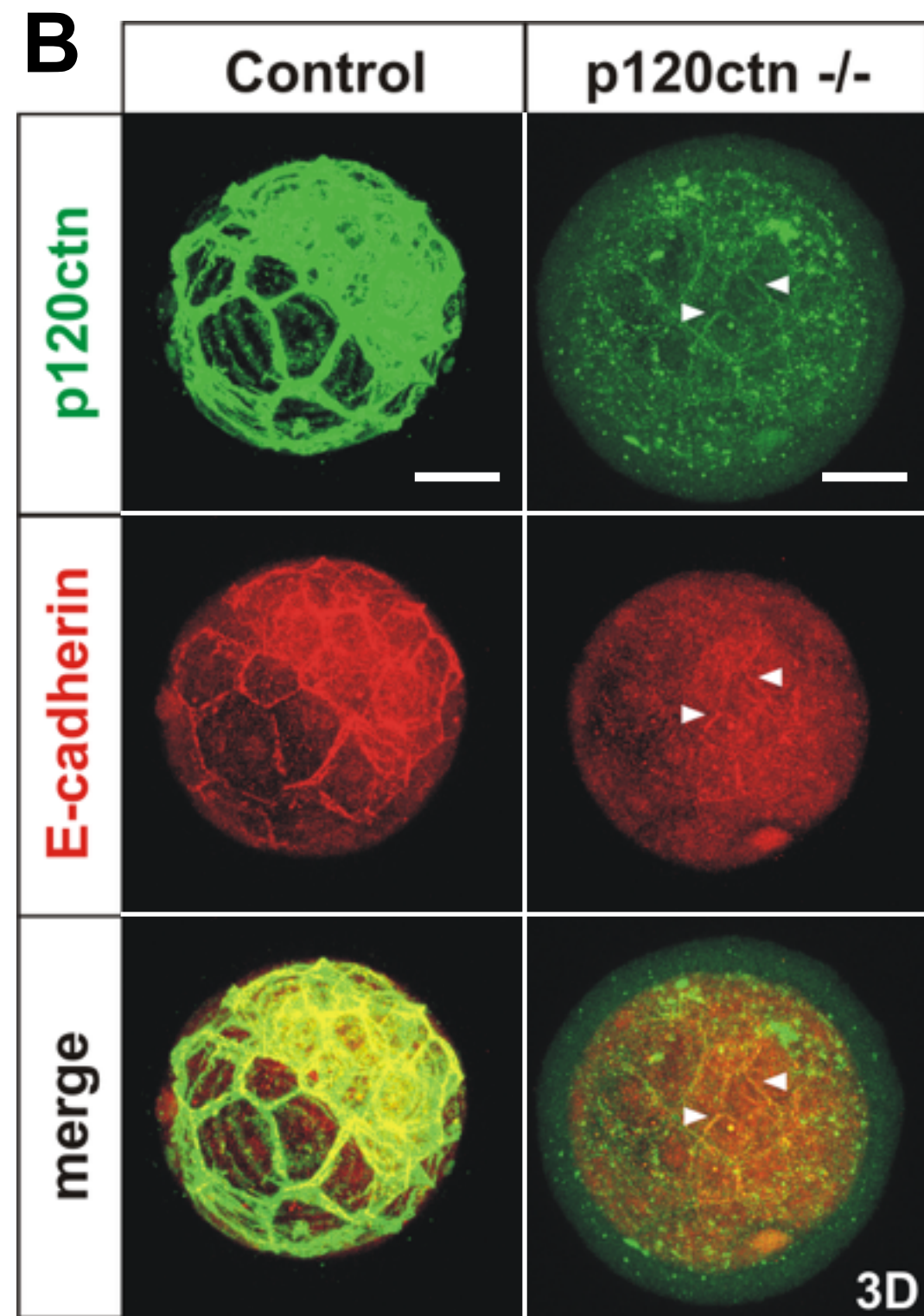

Supplement: S1 Fig — (A) Confocal fluorescent images of a wild-type blastocyst stained for p120ctn and the apical marker PKCζ. p120ctn is expressed basolaterally. Scale bar: 25 μm. (B) Maternal p120ctn allows basal E-cadherin stabilization on the membranes of p120ctn-deficient blastocysts. Three-dimensional (3D) reconstruction using consecutive confocal sections of the blastocysts shown in Fig 2E. The limited amount of maternal p120ctn in p120ctn-deficient embryos (p120ctn-/-, arrowheads) probably allows the stabilization of basal E-cadherin levels (arrowheads) on the membranes of blastocysts. This is thought to be sufficient for normal compaction and blastocyst formation. Scale bar: 25 μm. (PDF) [file pgen.1006243.s001.pdf]

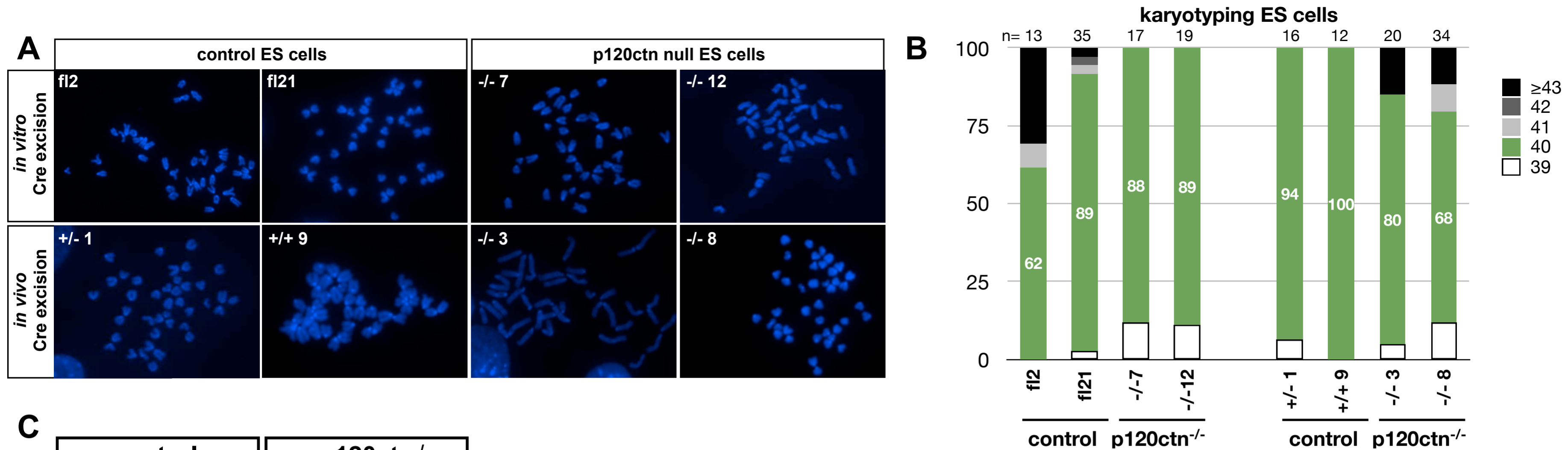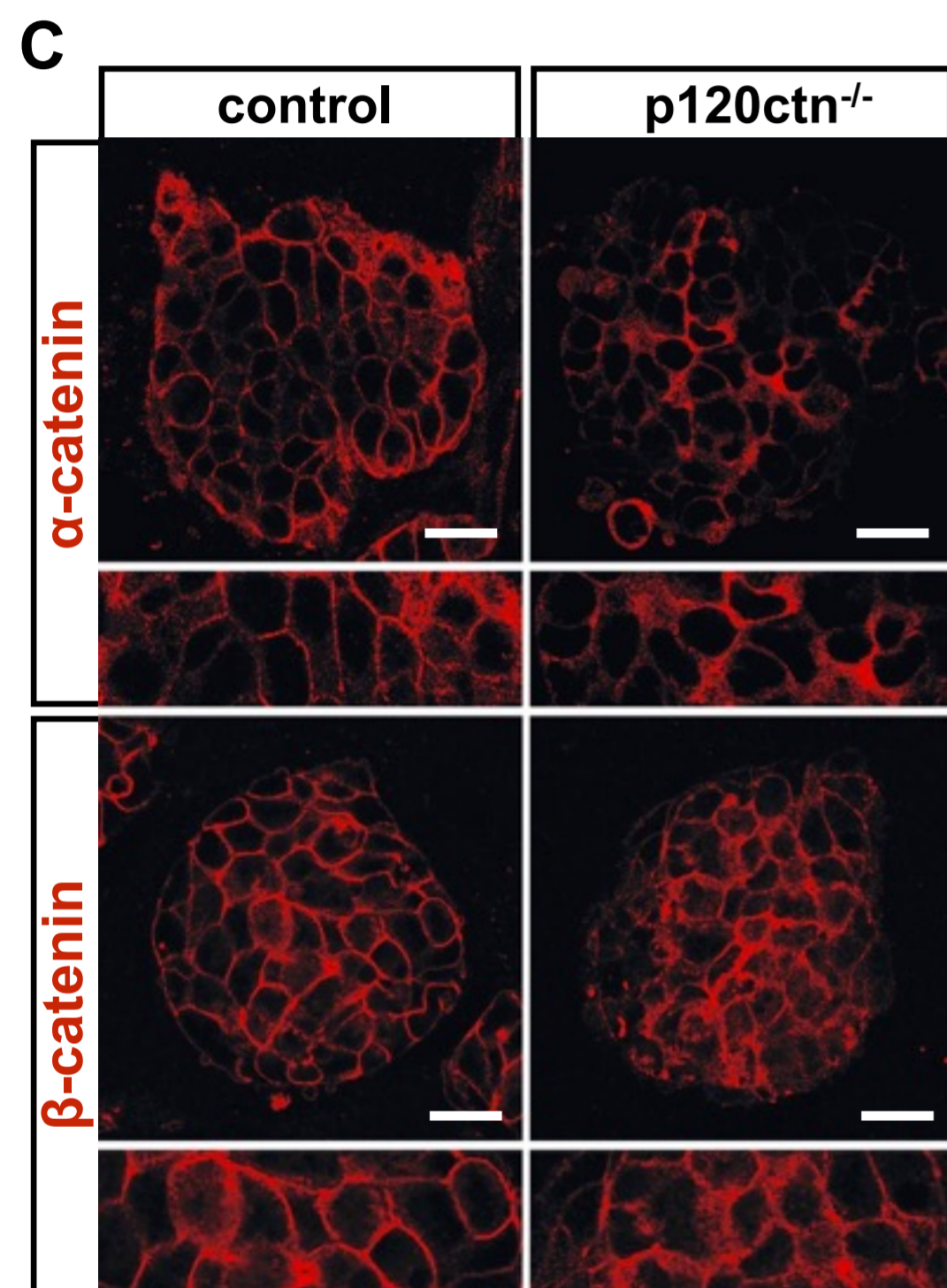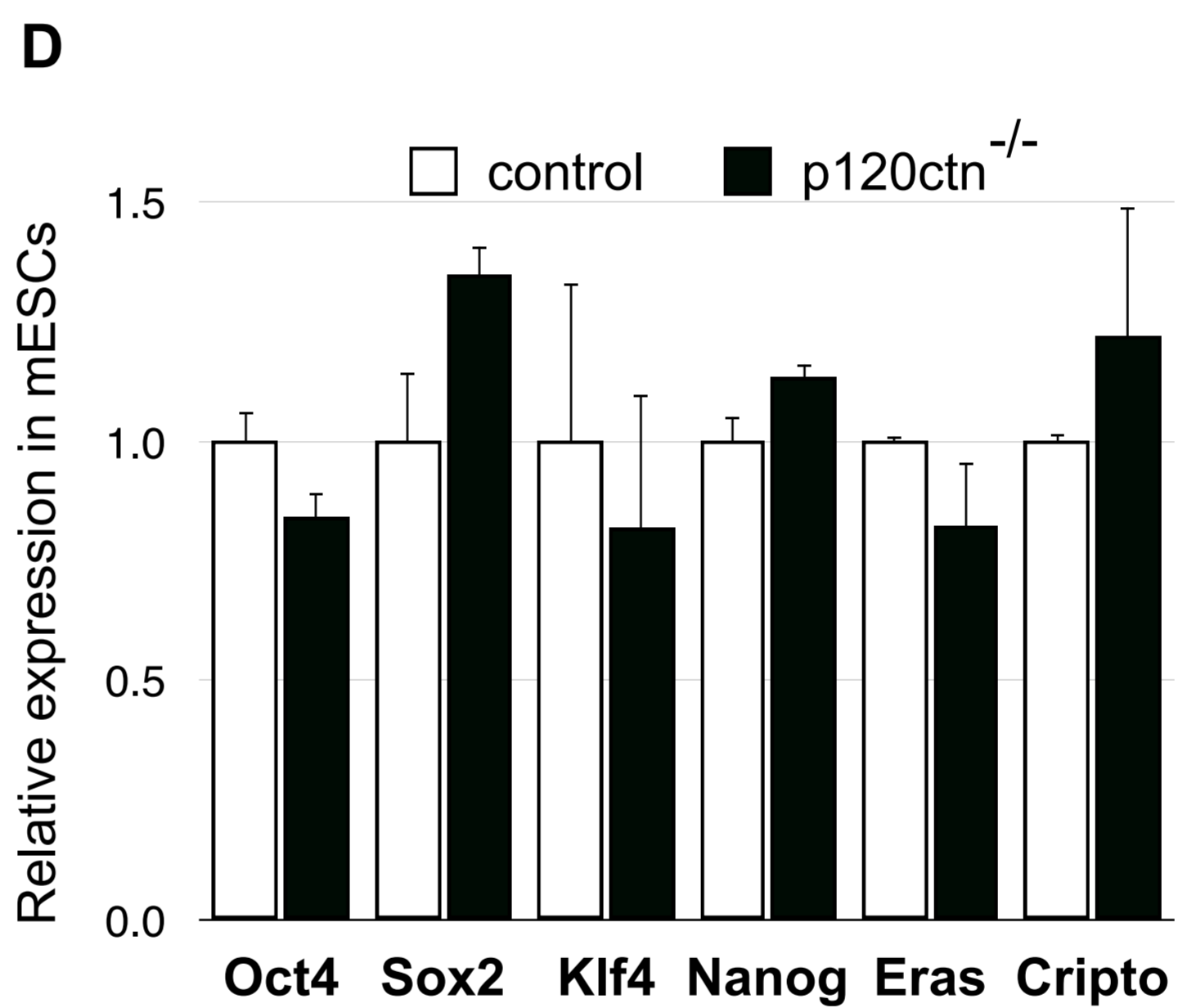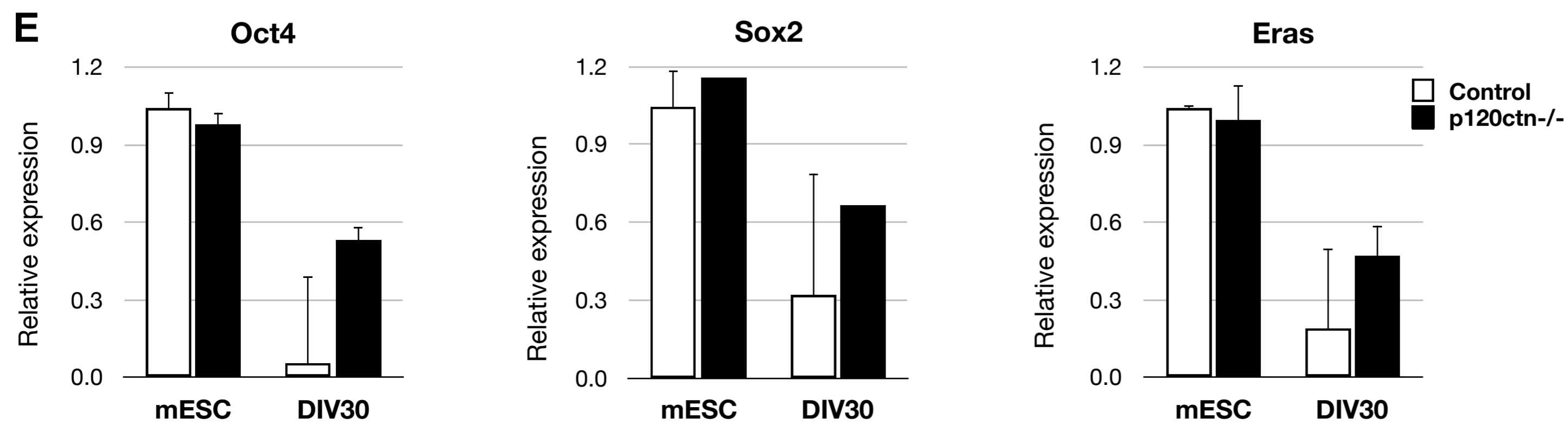

Supplement: S2 Fig — (A) Chromosomal analysis of control and p120ctn-null mESCs. Fluorescent images of DAPI-stained mitotic spreads with 40 acrocentric chromosomes from control mESC lines (p120ctnfl/fl (fl2, f21), p120ctn+/+ (+/+9), p120ctn+/- (+/-1)) and p120ctn-null mESCs (-/-7, -/-12, -/-3, -/-8). (B) Graph depicts the percentage of mitotic spreads that contain 39, 40, 41, 42 and 43 or more chromosomes. An mESC line is considered normal if 70% or more of its spreads contain 40 chromosomes. The names of mESC lines analyzed are at the bottom. (C) Confocal fluorescent images of control and p120ctn-null mESCs stained for α-catenin and β-catenin. The boxed areas were further magnified 3.6-fold. Scale bars: 25 μm. (D, E) qRT-PCR analysis for expression of various stemness genes, in (D) control and p120ctn-null mESCs, and in (E) their corresponding EBs after 30 days of culture (DIV30). Actb, Gapdh and Hmbs were used as reference genes. The error bars in the graphs represent the standard error of the mean of two independent control or p120ctn-null cell lines. (PDF) [file pgen.1006243.s002.pdf]

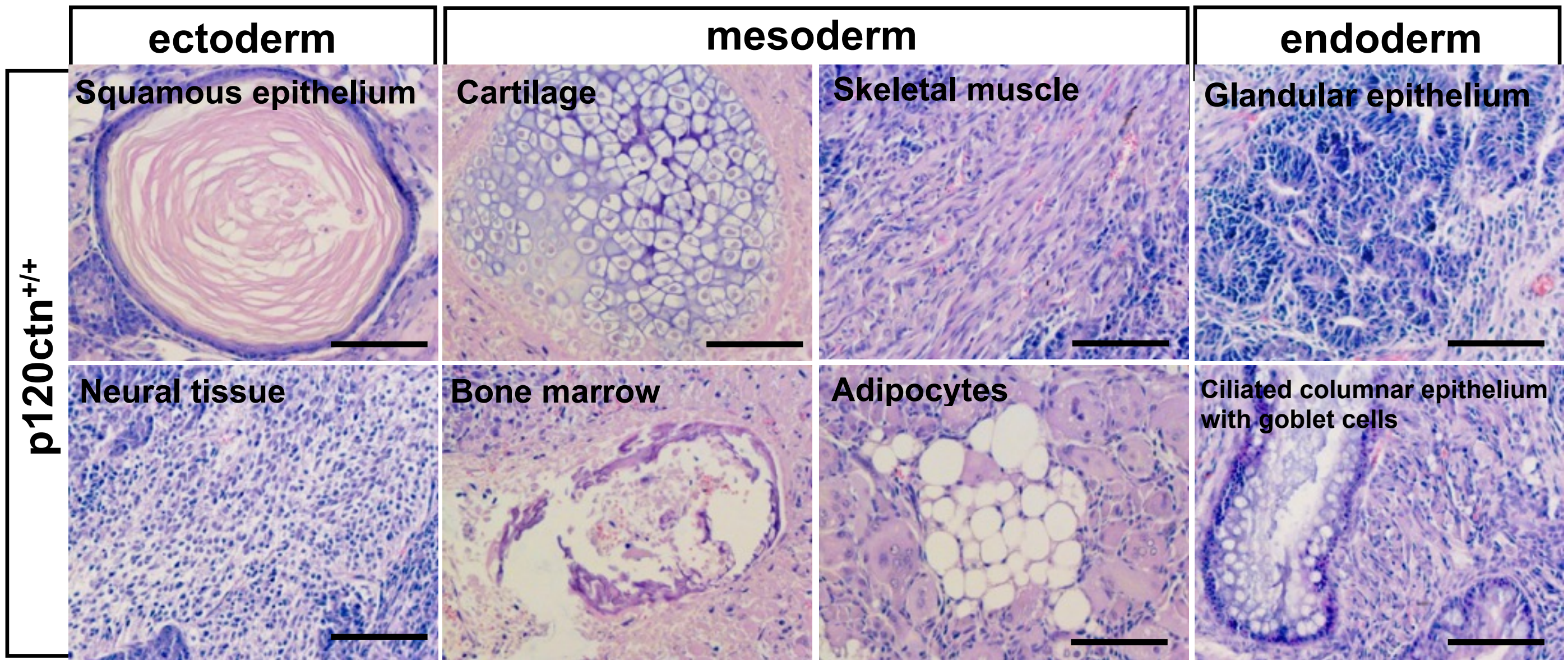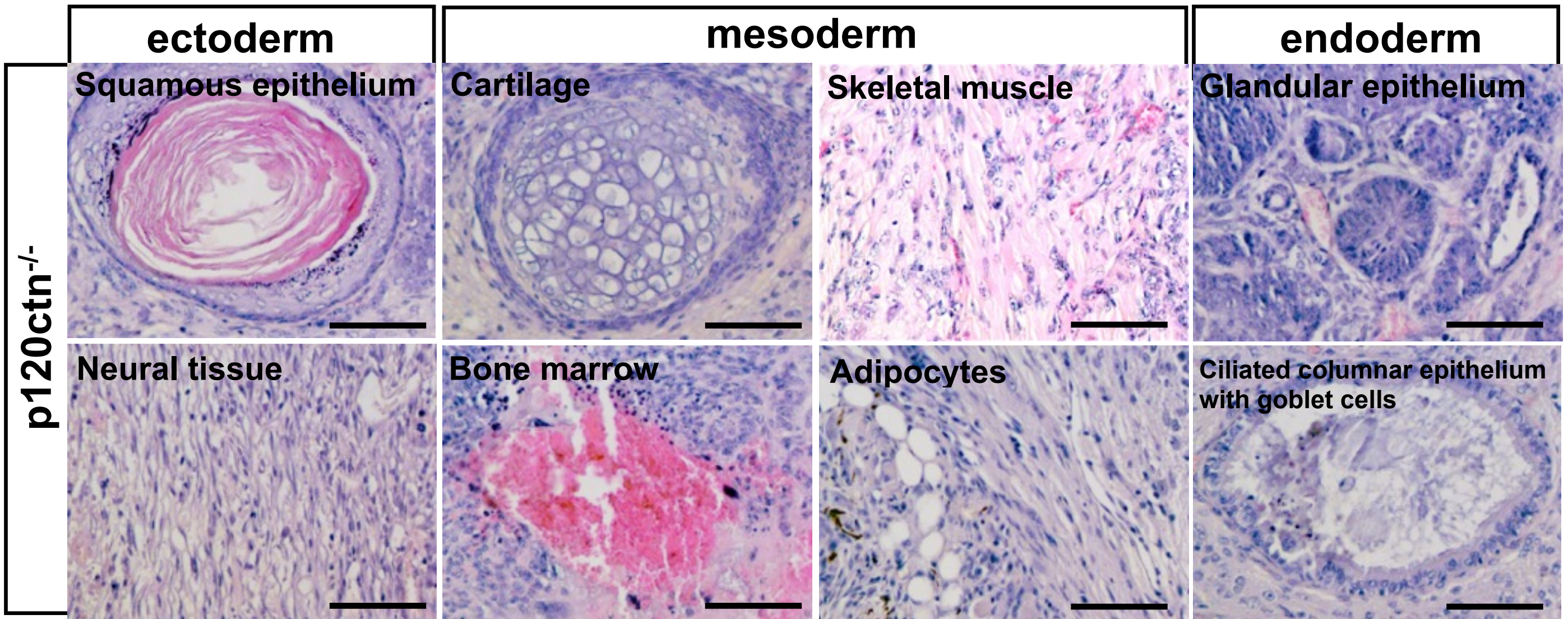

Supplement: S3 Fig — p120ctn loss does not abrogate germ layer development. Histological analysis of H&E stained sections of teratomas from control (p120ctn+/+) and p120ctn-depleted (p120ctn-/-) mESCs. Scale bars: 100 μm. (PDF) [file pgen.1006243.s003.pdf]

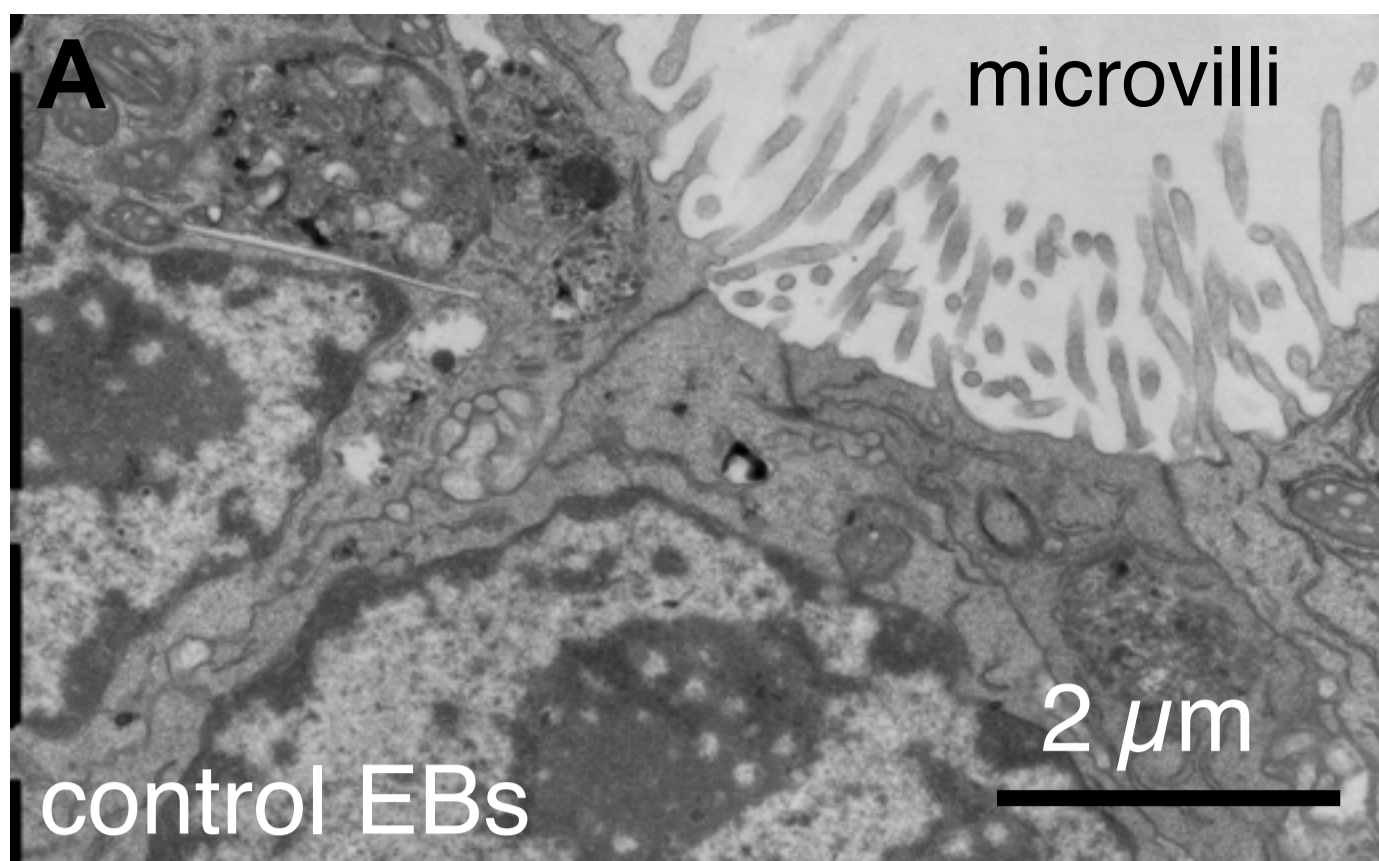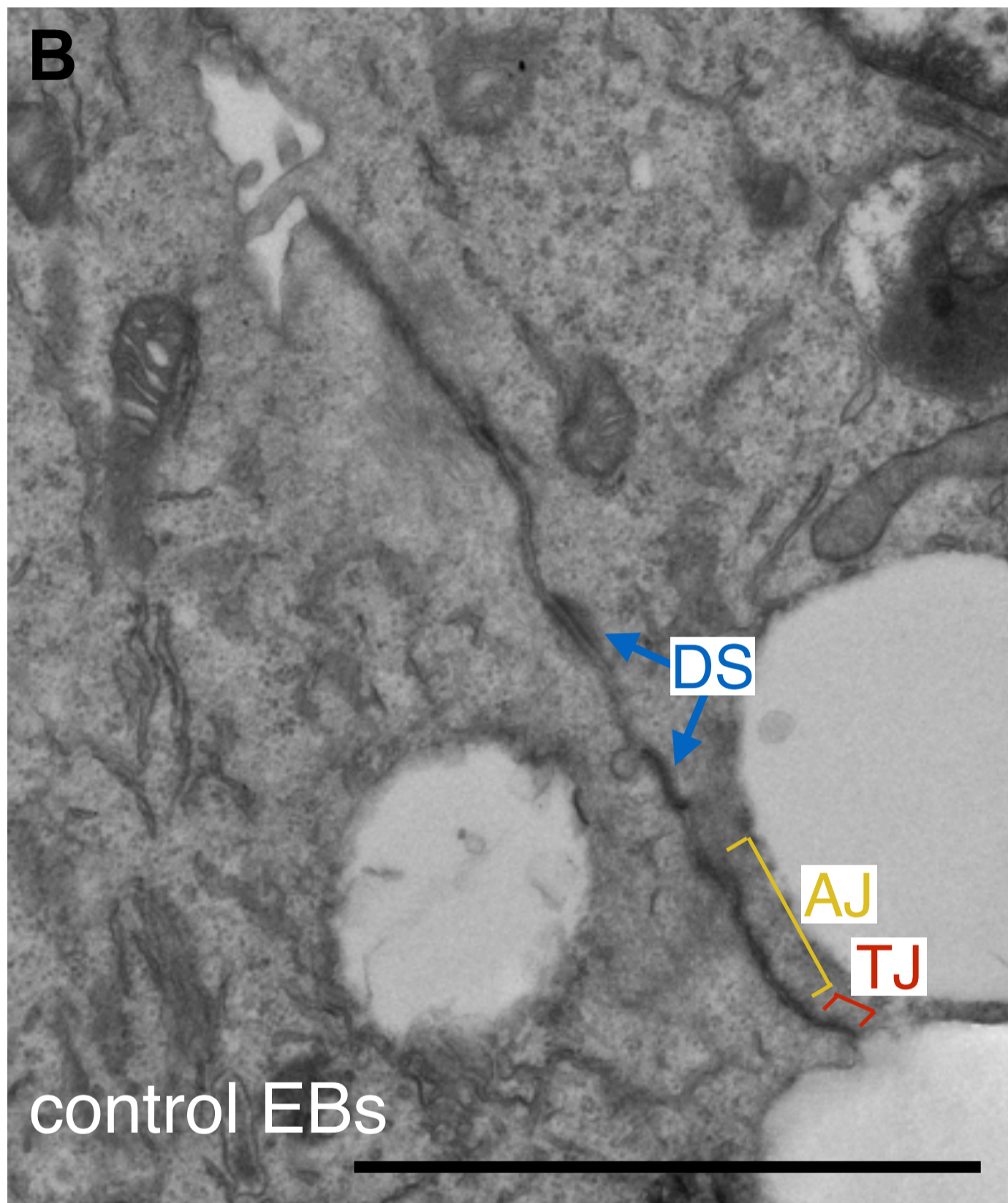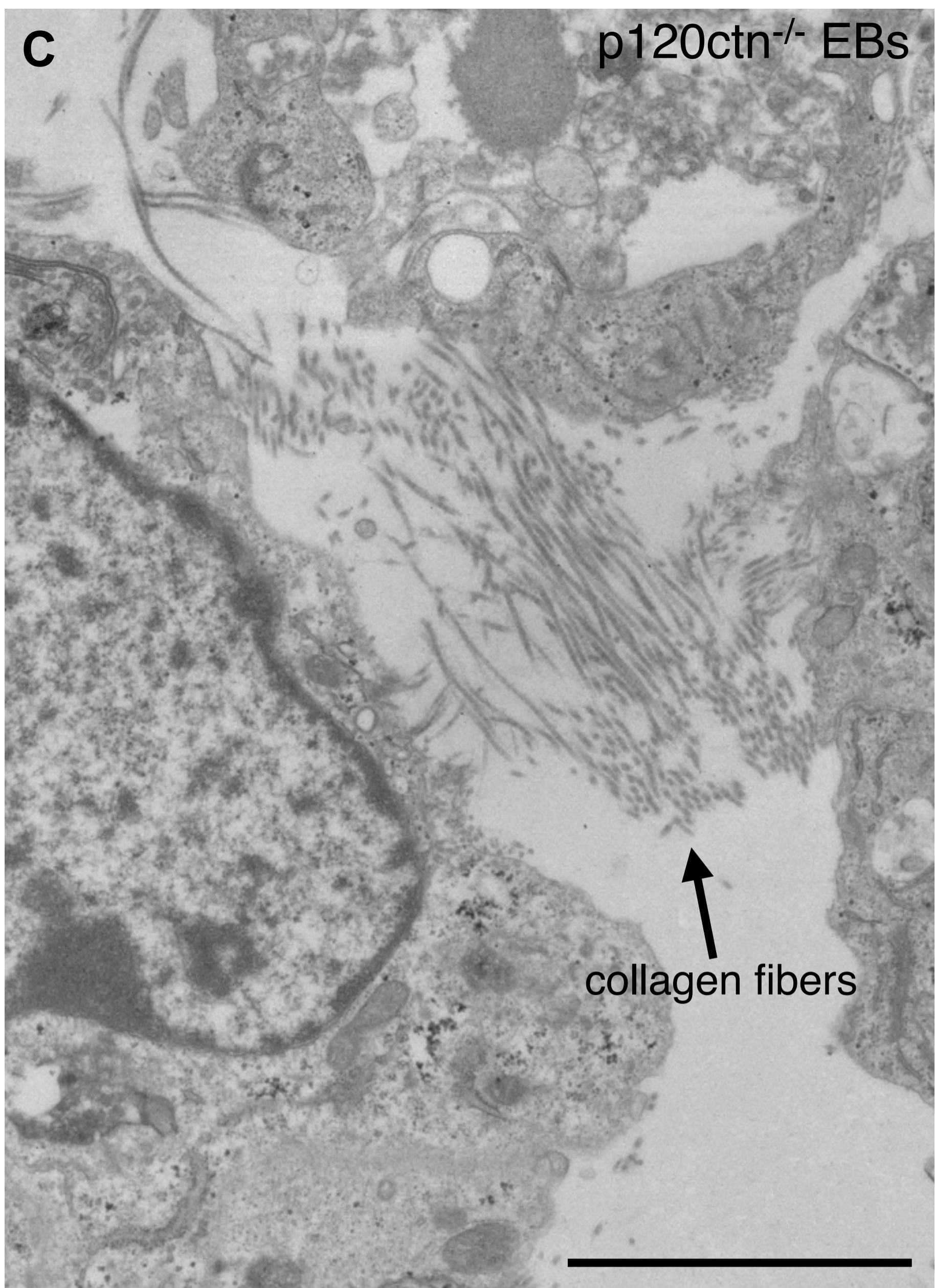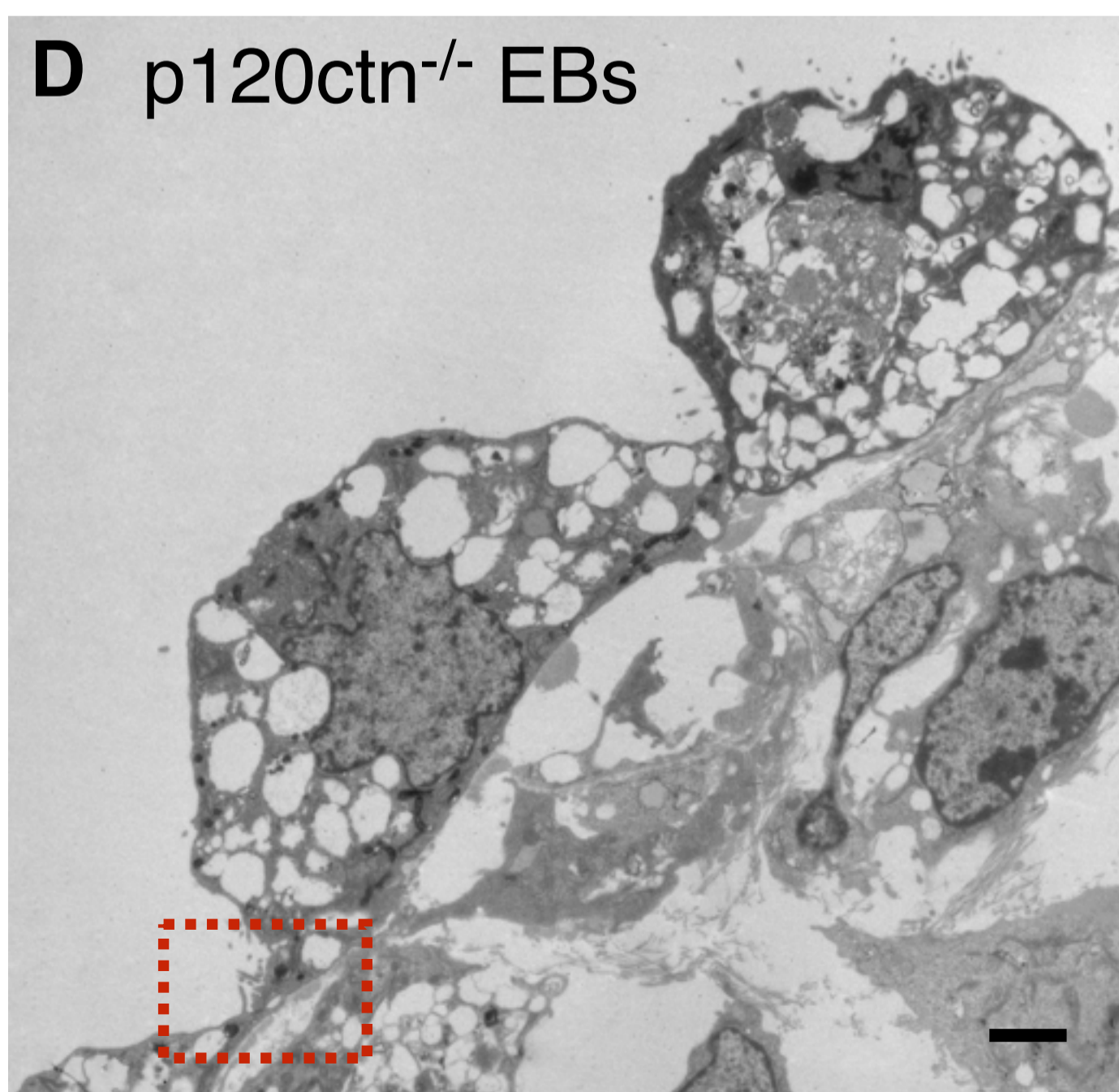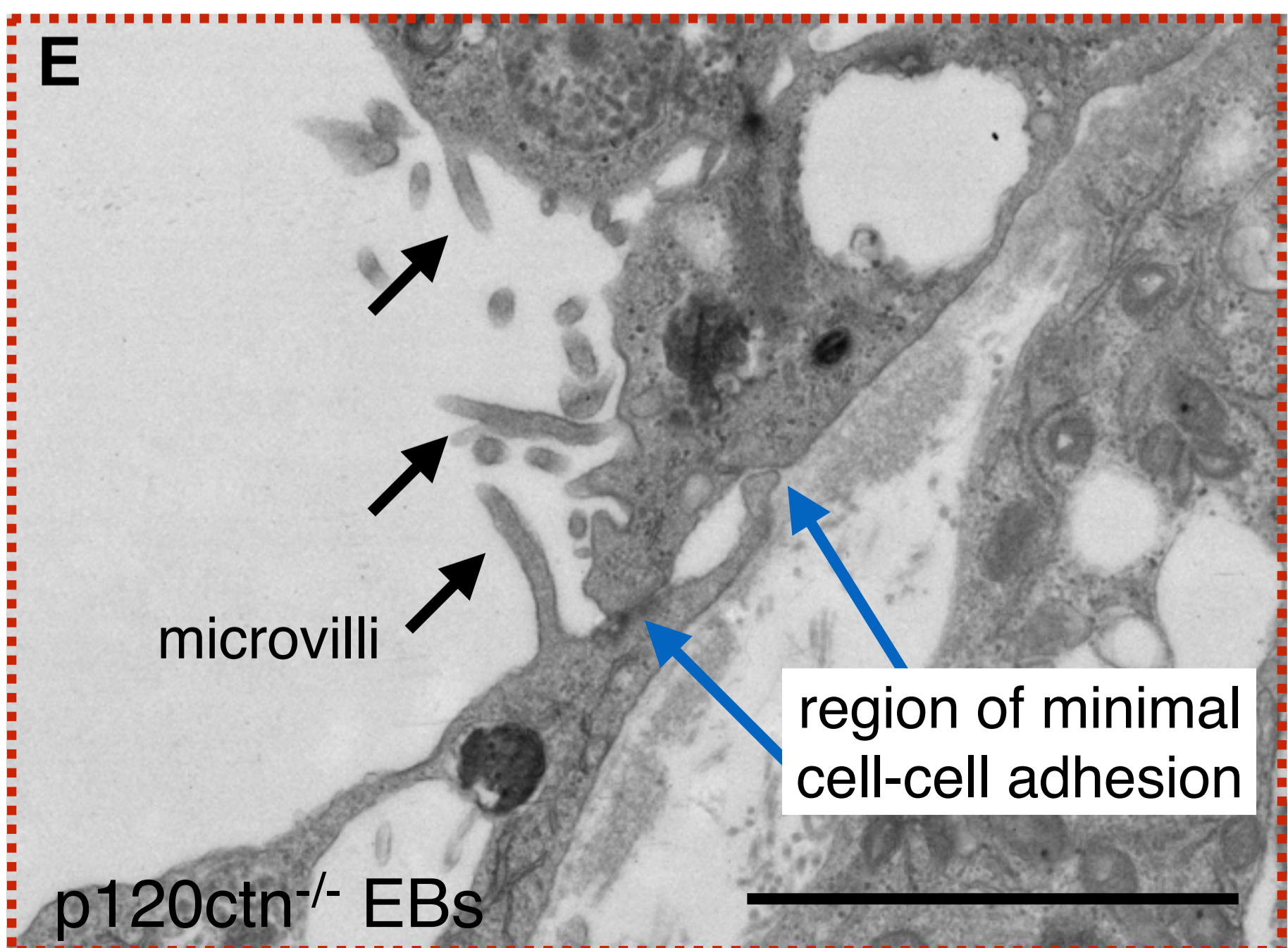

Supplement: S4 Fig — TEM analysis of DIV12 control EBs (A, B) and p120ctn-null EBs (C-E). The red dashed box in (D) is enlarged in (E) and shows a region of minimal endodermal cell-cell adhesion (blue arrows) showing non-polarized microvilli (black arrows). Scale bars: 2 μm. AJ, adherens junction; DS, desmosome; TJ, tight junction. (PDF) [file pgen.1006243.s004.pdf]

**A**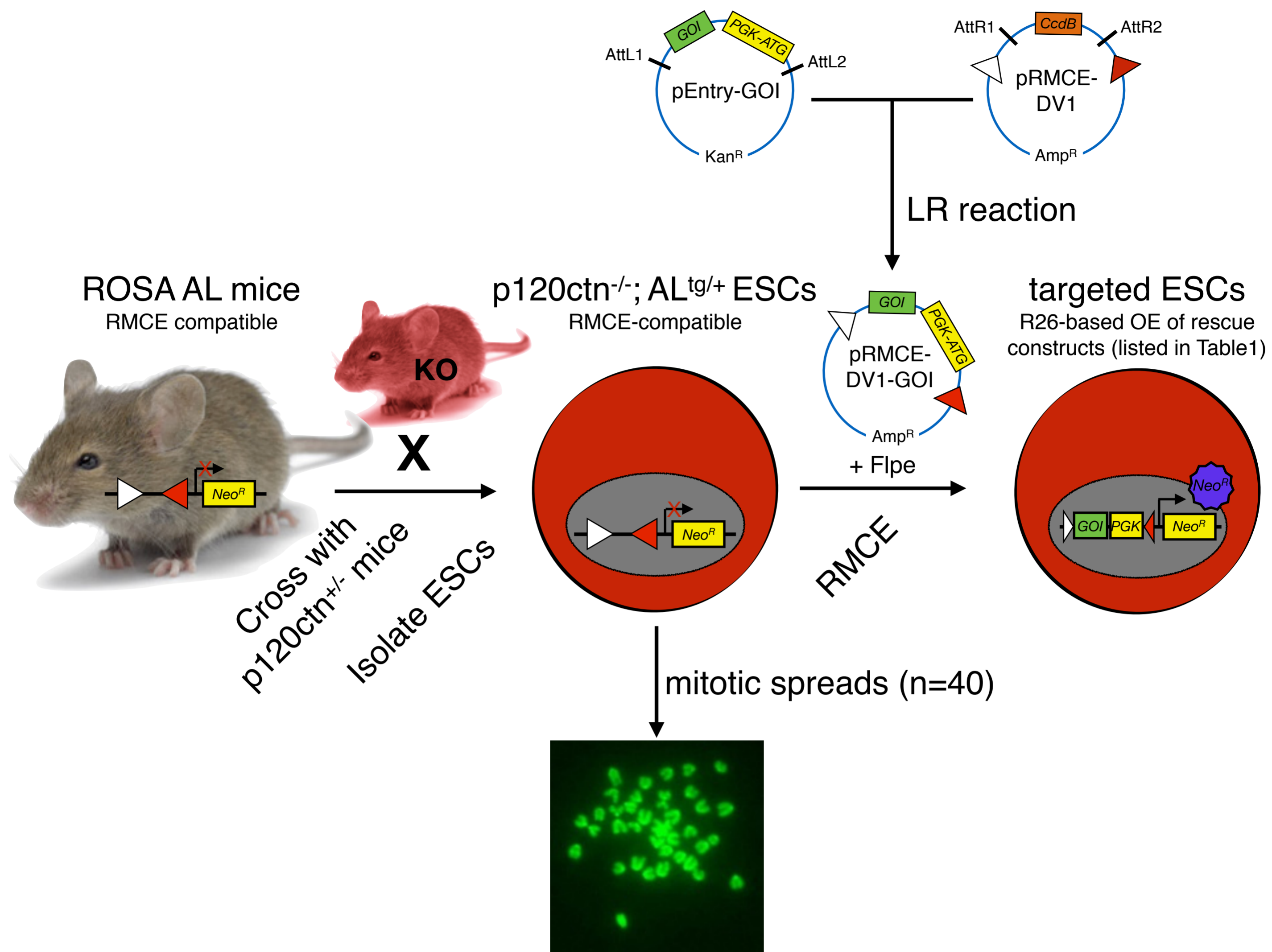**B**

**p120ctn levels in mESCs with R26-driven rescue constructs**

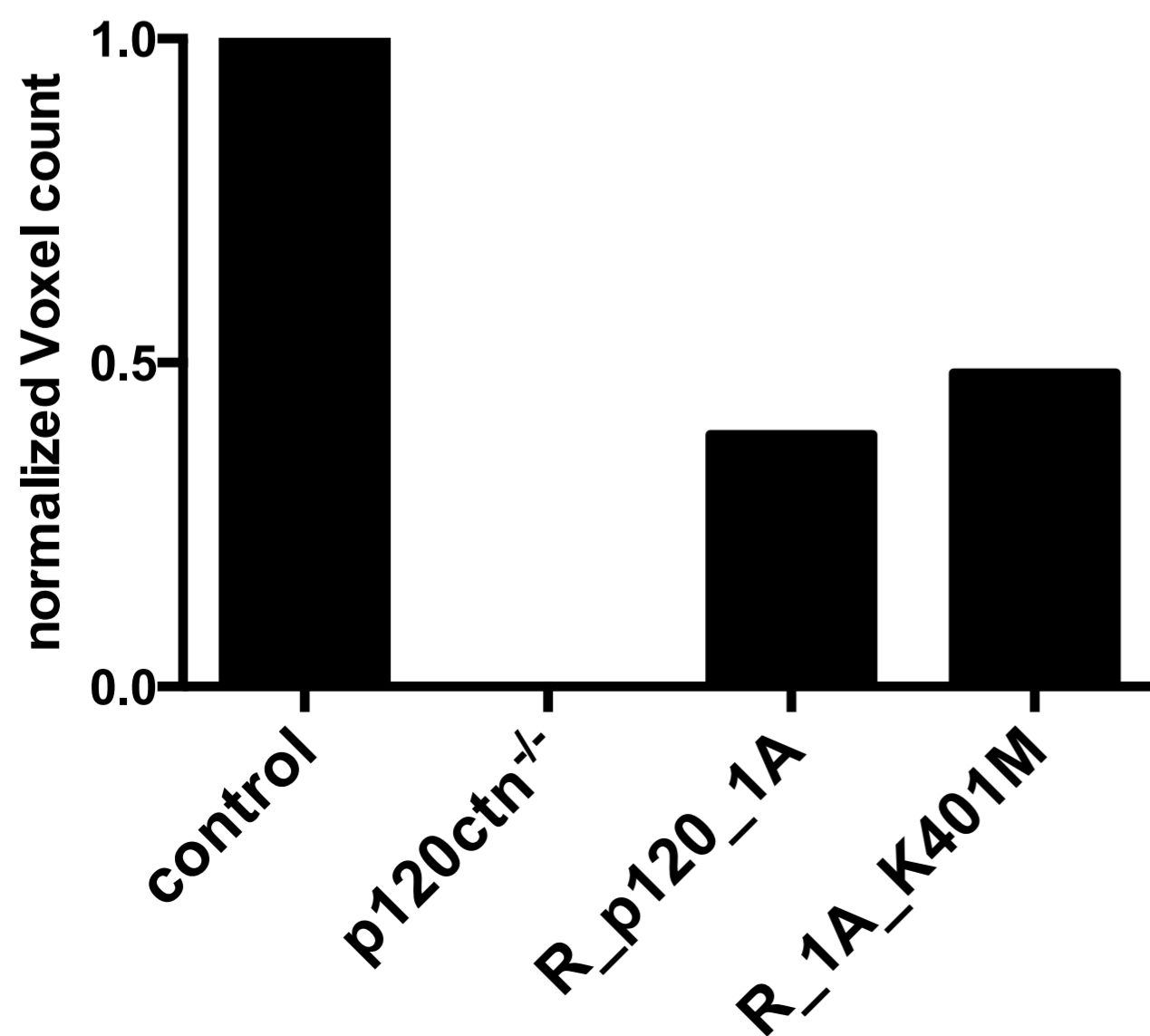**C**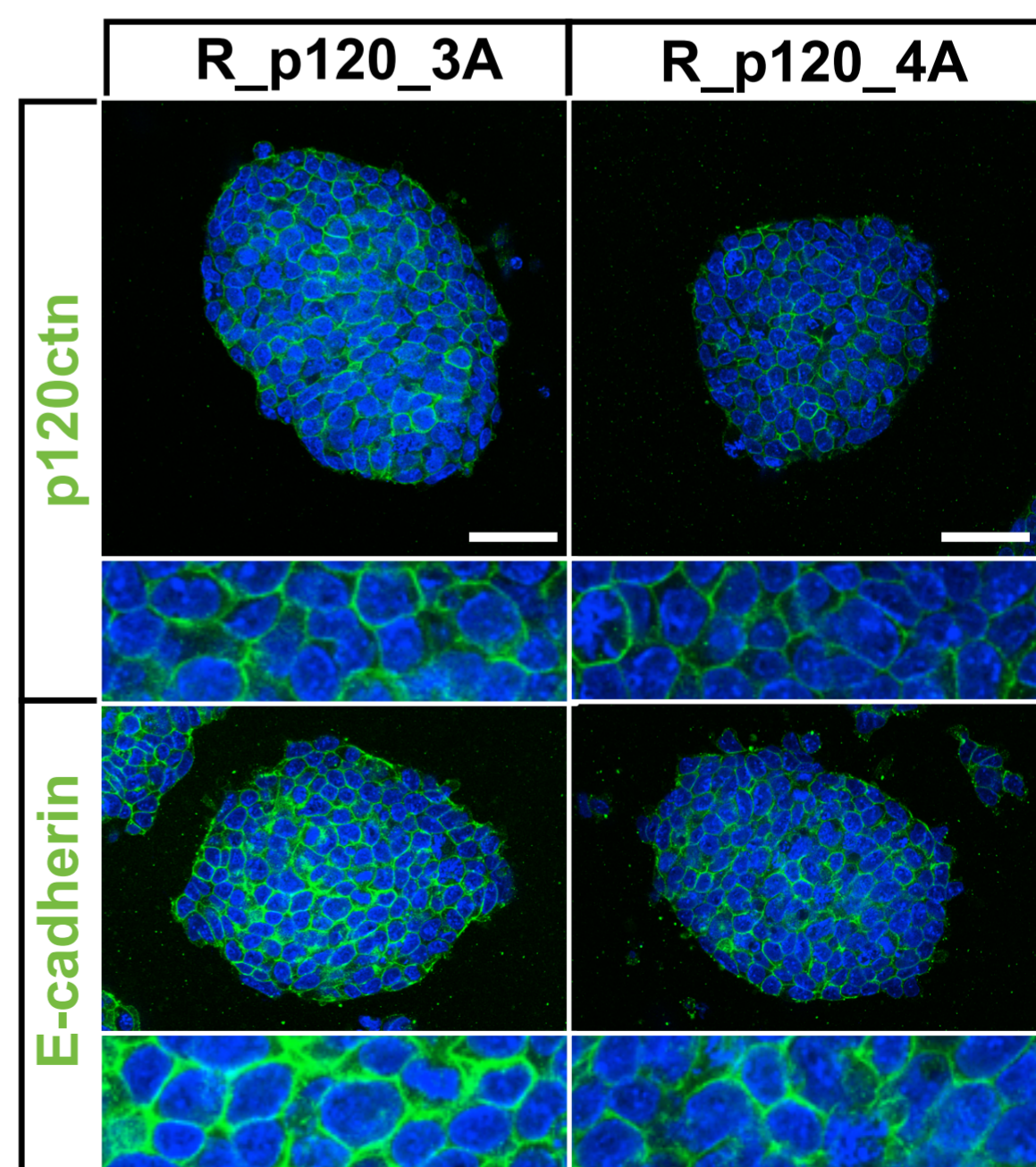

Supplement: S5 Fig — (A) Scheme depicting our mouse breeding protocol, followed by isolation of the RMCE-compatible p120ctn-/-;ALtg/+ mESCs, and insertion of various rescue cDNAs in the ROSA26 locus by RMCE. By Gateway cloning we inserted a set of candidate rescue cDNAs (listed in Table 1) into an RMCE-compatible destination vector, called pRMCE-DV1, which also harbors two heterospecific Frt sites, which do not cross-react with each other (depicted by white and red triangles), followed by a PGK promoter and the start codon of the NeoR gene [71]. We co-transfected p120ctn-/-;ALtg/+ mESCs with the different pRMCE-DV1 plasmids and with a Flpe expression plasmid. Flpe-mediated cassette exchange inserted the gene of interest (GOI) into the ROSA26 locus and in addition restored neomycin-resistance. In these targeted mESCs, both the GOI and NeoR genes are driven by the endogenous R26 promoter. A fluorescent image of a DAPI-stained mitotic spread with 40 acrocentric chromosomes from p120ctn-/-;ALtg/+ mESCs is shown at the bottom. (B) Graph depicting p120ctn levels in control and p120ctn-null mESC, and in p120ctn-null mESC with R26-driven expression of p120ctn isoform 1A (R_p120_1A) or of its K401M mutant (R_1A_K401M). Z-stacks, optimized according to the Nyquist sampling theorem, were acquired on the SP5 Leica confocal microscope. A fixed intensity threshold was set on the Alexa 488 signal representing p120ctn staining. Within this threshold, the total amount of voxels for each mESC colony was counted and normalized against its total nuclear volume. At least 10 reconstructed colonies were analyzed for each mESC line. (C) Confocal fluorescent images of p120ctn-null mESCs with R26-driven expression of p120ctn isoform 3A (R_p120_3A) or 4A (R_p120_4A) stained for p120ctn or E-cadherin expression. A threefold magnified image is shown below each picture. Scale bars: 50 μm. (PDF) [file pgen.1006243.s005.pdf]

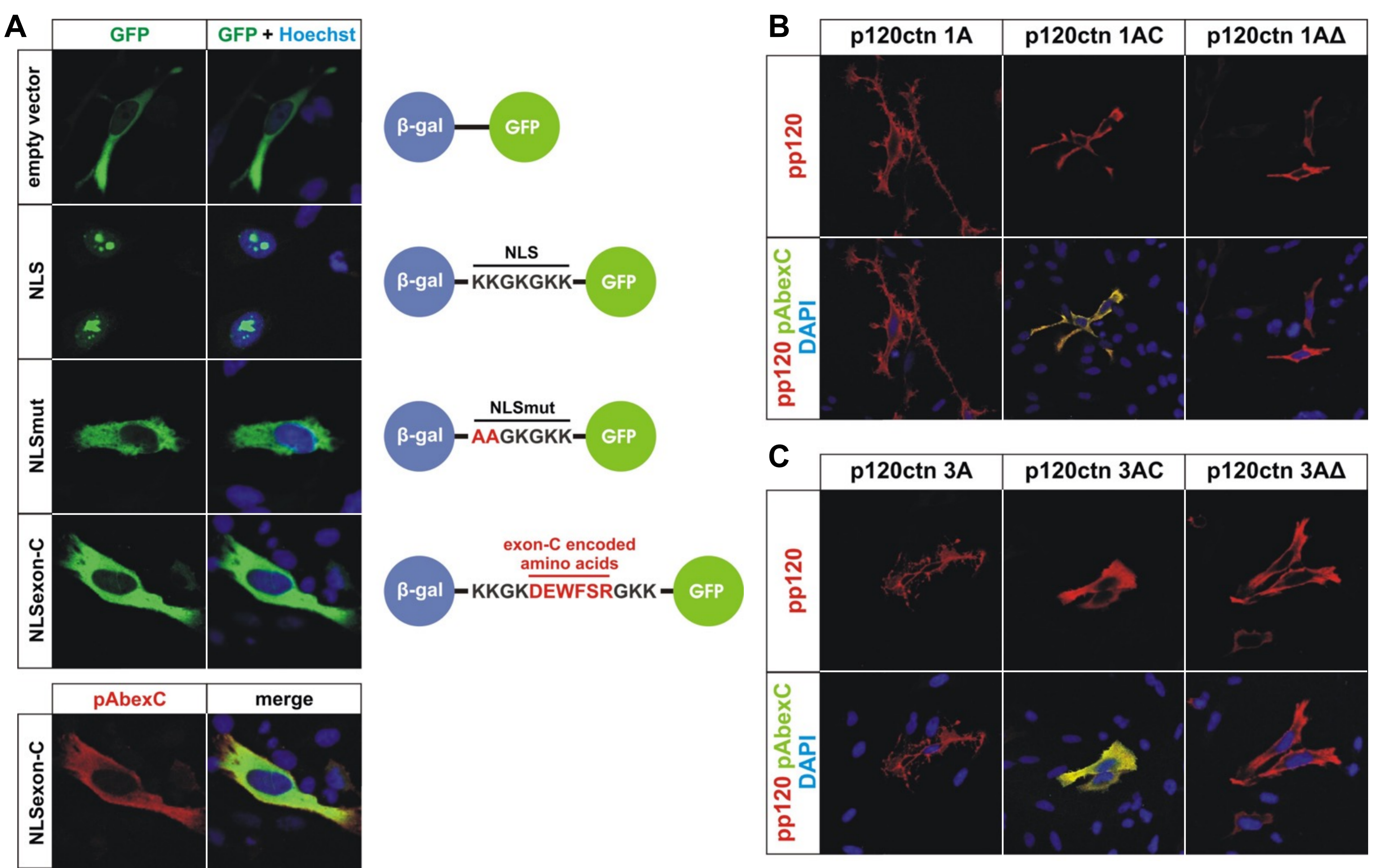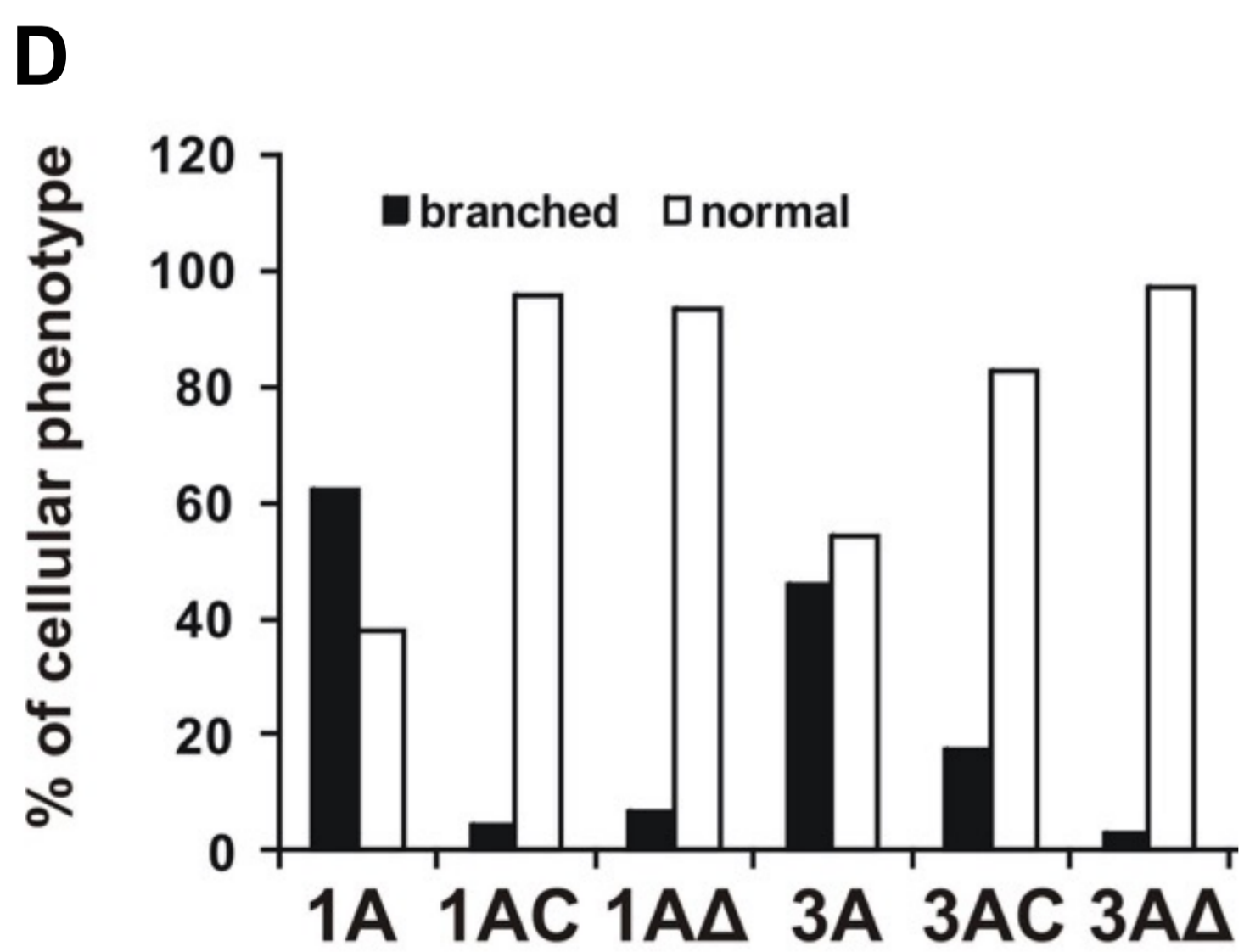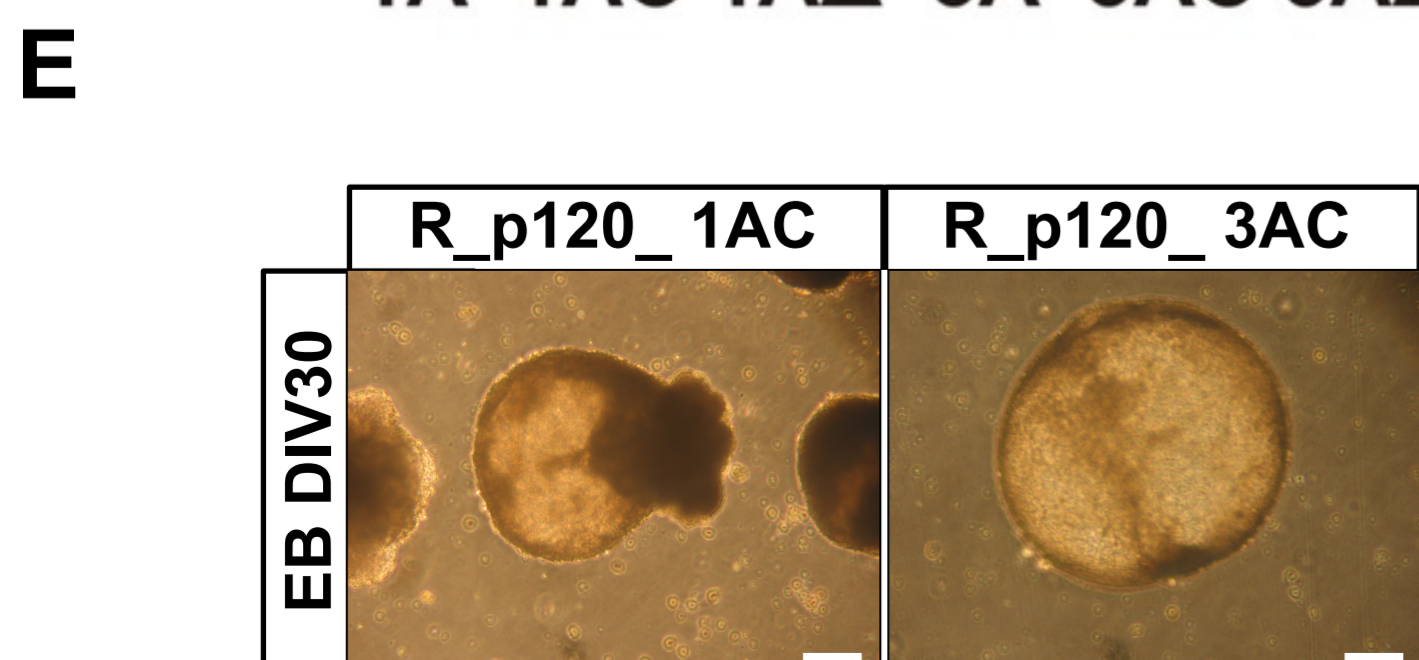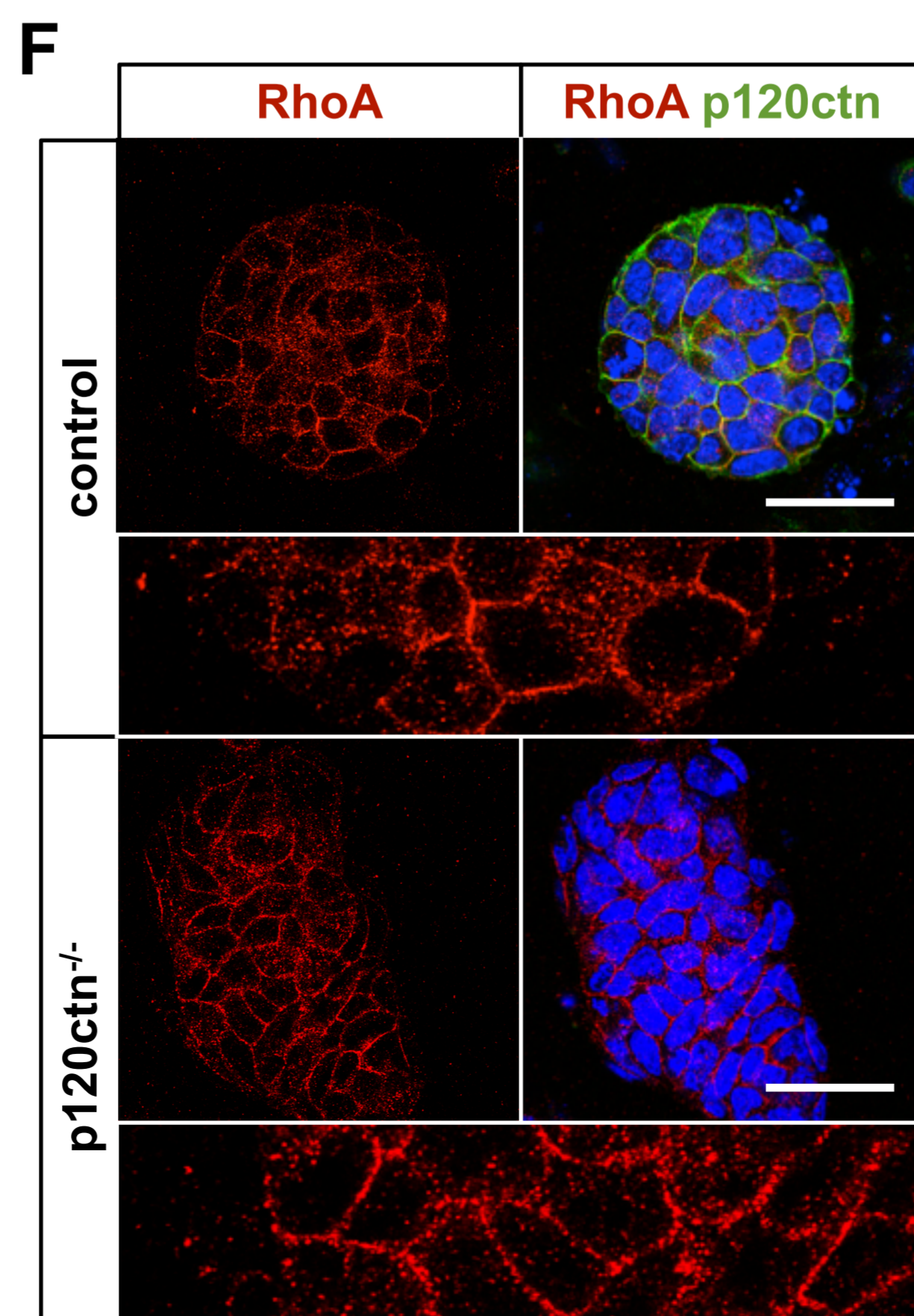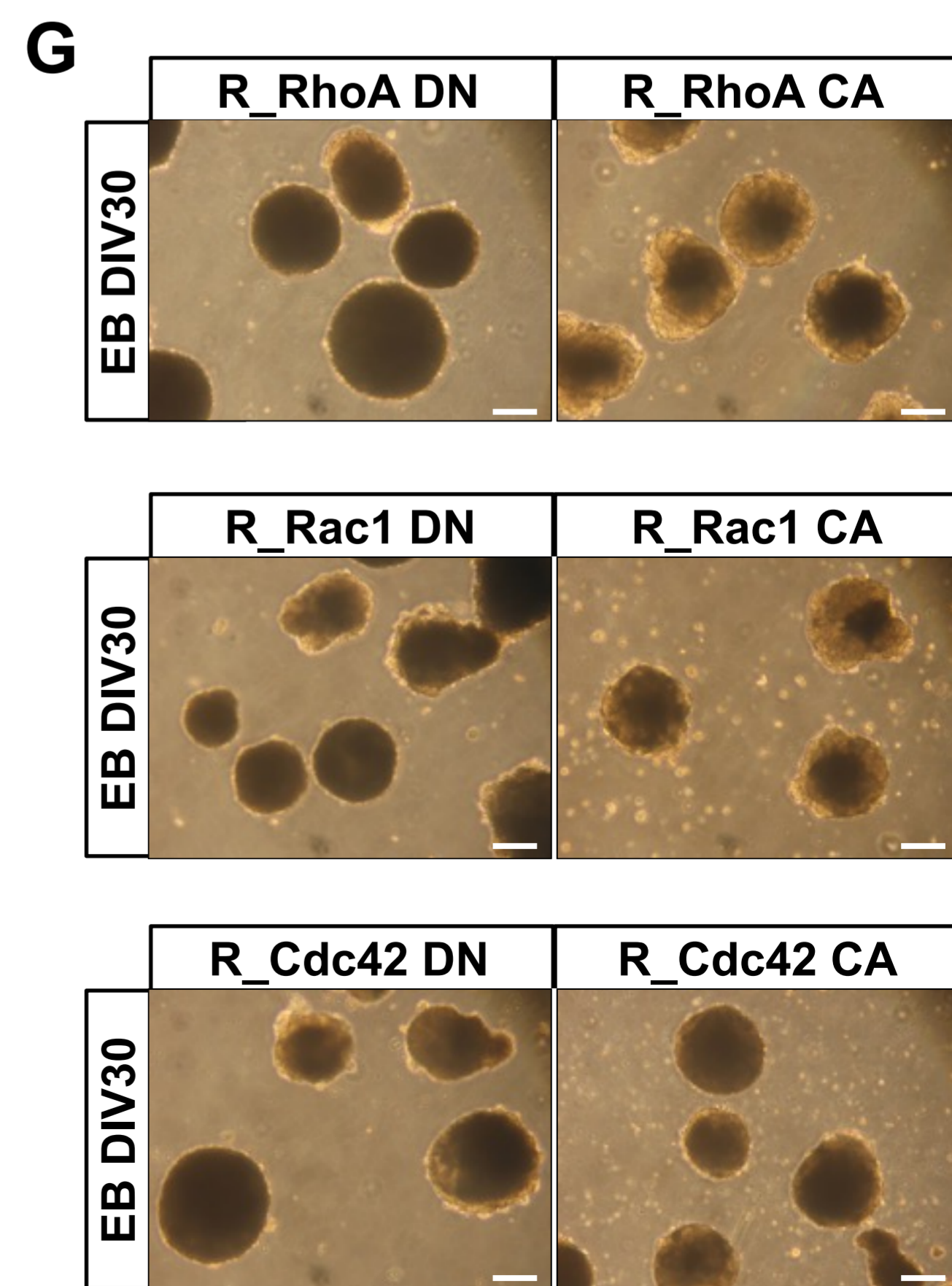

Supplement: S6 Fig — Amino acids encoded by p120ctn exon-C inhibit nuclear translocation and dendritic-like branching. (A) Nuclear translocation assay using fusion proteins composed of an N-terminal β-galactosidase (β-gal) part and a C-terminal GFP. Between the β-gal and the GFP parts we cloned the NLS of p120ctn (NLS, AA622-628), a mutated version of it (NLSmut), or the NLS interrupted by amino acids encoded by exon-C (NLSexon-C). These constructs were expressed in HeLa cells. Confocal fluorescence analysis showed that both NLSmut and NLSexon-C prevented the nuclear GFP expression seen with the NLS construct. The exon-C encoded AA expressed by the NLSexon-C construct were also specifically detected by an in-house made polyclonal antibody (pAb exC, bottom panel). (B, C) Branching assay in HeLa cells transiently transfected with plasmids expressing either (B) p120ctn isoform 1A and related proteins, or (C) isoform 3A and related proteins. The related proteins were p120ctn isoform C variants (p120ctn isoforms 1AC and 3AC), or mutants lacking amino acids 622–628 (p120ctn isoforms 1AΔ and 3AΔ). Confocal images were made after immunostaining for all p120ctn isoforms (pp120, red), either combined or not combined with specific staining for p120ctn isoform C (pAb exC, green). (D) Graph showing the percentage of branched versus normal cellular phenotypes upon overexpression of different p120ctn isoforms in HeLa cells, as illustrated in panels (B) and (C). (E) Micrographs of DIV30 p120ctn-null EBs with R26-driven expression of p120ctn isoform 1AC (R_p120_1AC) or 3AC (R_p120_3AC). Two independently rescued mESC lines were analyzed in each setup. Scale bars: 200 μm. (F) Confocal fluorescent pictures of control and p120ctn-null mESCs stained for p120ctn (green) and RhoA (red). A 3.3-fold magnification is shown below each picture. Scale bars: 15 μm. (G) Micrographs of DIV30 p120ctn-null EBs with R26-driven expression of dominant-negative (DN) or constitutively active (CA) Rho GTPases, as indicated. T [file pgen.1006243.s006.pdf]

**A**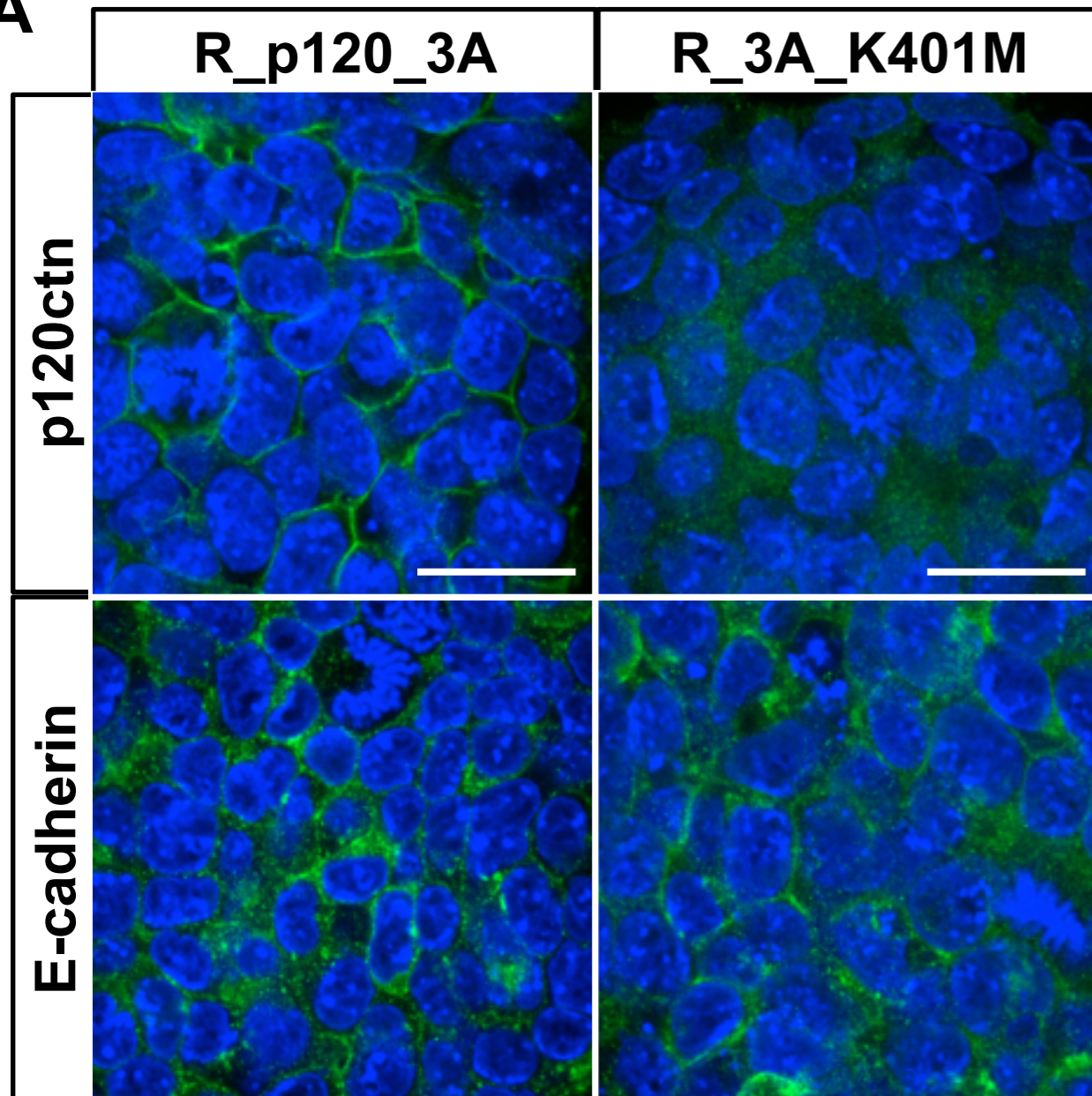**B**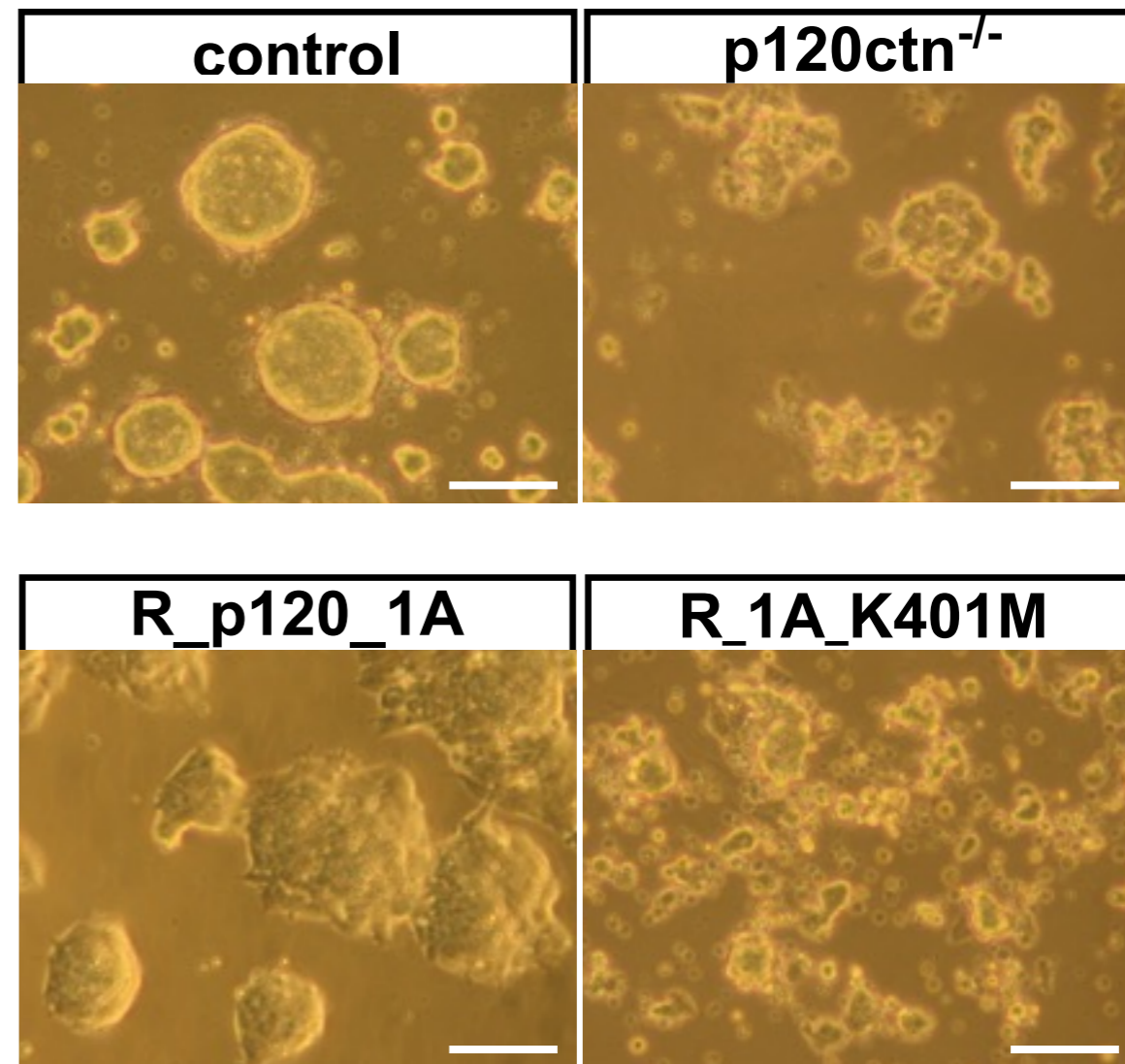

Supplement: S7 Fig — (A) Confocal fluorescent pictures of R_p120_3A mESCs, and of mESCs expressing the K401M mutant of p120ctn isoform 3, after immunostaining for p120ctn and E-cadherin. Scale bars: 25 μm. (B) Micrographs depicting the morphology of different mESC cultures, as indicated. Scale bars: 100 μm (PDF) [file pgen.1006243.s007.pdf]

**A**

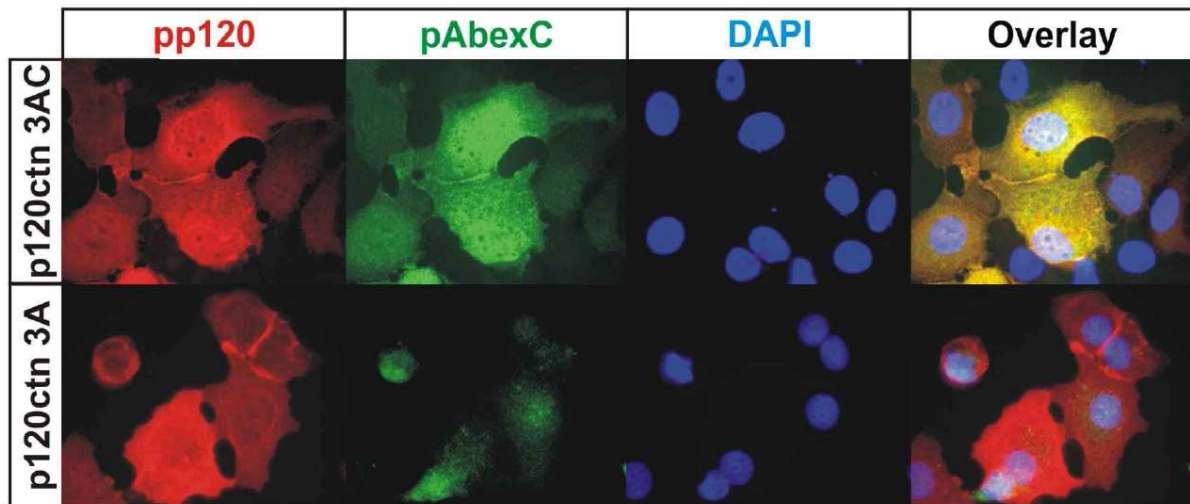

**B**

mouse      7,5 dpc  
brain      embryos

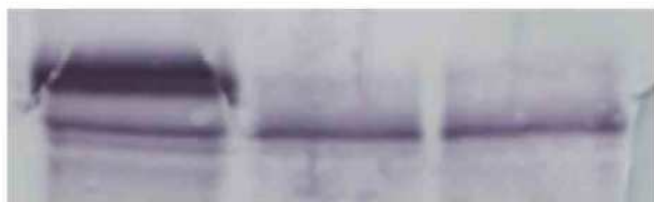

- 100 kDa

WB: pp120

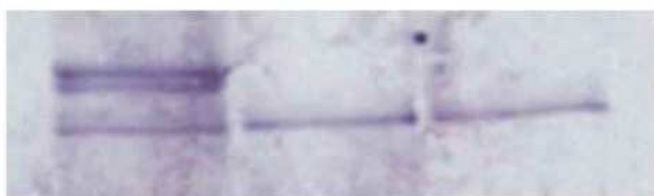

- 100 kDa

WB:pAbexC

Supplement: S8 Fig — (A) Immunostaining to characterize an antibody specific for isoform C of p120ctn (pAb ExC). This antibody recognizes in transiently transfected MCF7 cells human p120ctn isoform 3AC, but not human isoform 3A lacking exon-C encoded amino acids. Monoclonal antibody pp120 recognizes all p120 isoforms. (B) Western blot analysis of two wild-type gastrulating mouse embryos (E7.5) in which all endogenous p120ctn isoforms were detected with pp120 antibody and endogenous p120ctn isoforms C were detected with pAb ExC. Mouse brain was taken as a positive control because it is relatively rich in isoforms C of p120ctn. (PDF) [file pgen.1006243.s008.pdf]
